# Blasticidin S inhibits mammalian translation and enhances production of protein encoded by nonsense mRNA

**DOI:** 10.1093/nar/gkab532

**Published:** 2021-06-22

**Authors:** Kyle T Powers, Flint Stevenson-Jones, Sathish K N Yadav, Beate Amthor, Joshua C Bufton, Ufuk Borucu, Dakang Shen, Jonas P Becker, Daria Lavysh, Matthias W Hentze, Andreas E Kulozik, Gabriele Neu-Yilik, Christiane Schaffitzel

**Affiliations:** University of Bristol, School of Biochemistry, University Walk, Bristol BS8 1TD, UK; University of Bristol, School of Biochemistry, University Walk, Bristol BS8 1TD, UK; University of Bristol, School of Biochemistry, University Walk, Bristol BS8 1TD, UK; Department of Pediatric Oncology, Hematology and Immunology, Hopp Children's Cancer Research Center Heidelberg (KiTZ), University of Heidelberg, Heidelberg, Germany; Molecular Medicine Partnership Unit (MMPU) European Molecular Biology Laboratory (EMBL) and University of Heidelberg, Heidelberg, Germany; University of Bristol, School of Biochemistry, University Walk, Bristol BS8 1TD, UK; University of Bristol, School of Biochemistry, University Walk, Bristol BS8 1TD, UK; University of Bristol, School of Biochemistry, University Walk, Bristol BS8 1TD, UK; Department of Pediatric Oncology, Hematology and Immunology, Hopp Children's Cancer Research Center Heidelberg (KiTZ), University of Heidelberg, Heidelberg, Germany; Molecular Medicine Partnership Unit (MMPU) European Molecular Biology Laboratory (EMBL) and University of Heidelberg, Heidelberg, Germany; Department of Pediatric Oncology, Hematology and Immunology, Hopp Children's Cancer Research Center Heidelberg (KiTZ), University of Heidelberg, Heidelberg, Germany; Molecular Medicine Partnership Unit (MMPU) European Molecular Biology Laboratory (EMBL) and University of Heidelberg, Heidelberg, Germany; Molecular Medicine Partnership Unit (MMPU) European Molecular Biology Laboratory (EMBL) and University of Heidelberg, Heidelberg, Germany; European Molecular Biology Laboratory (EMBL), Heidelberg, Germany; Department of Pediatric Oncology, Hematology and Immunology, Hopp Children's Cancer Research Center Heidelberg (KiTZ), University of Heidelberg, Heidelberg, Germany; Molecular Medicine Partnership Unit (MMPU) European Molecular Biology Laboratory (EMBL) and University of Heidelberg, Heidelberg, Germany; Department of Pediatric Oncology, Hematology and Immunology, Hopp Children's Cancer Research Center Heidelberg (KiTZ), University of Heidelberg, Heidelberg, Germany; Molecular Medicine Partnership Unit (MMPU) European Molecular Biology Laboratory (EMBL) and University of Heidelberg, Heidelberg, Germany; University of Bristol, School of Biochemistry, University Walk, Bristol BS8 1TD, UK

## Abstract

Deciphering translation is of paramount importance for the understanding of many diseases, and antibiotics played a pivotal role in this endeavour. Blasticidin S (BlaS) targets translation by binding to the peptidyl transferase center of the large ribosomal subunit. Using biochemical, structural and cellular approaches, we show here that BlaS inhibits both translation elongation and termination in Mammalia. Bound to mammalian terminating ribosomes, BlaS distorts the 3′CCA tail of the P-site tRNA to a larger extent than previously reported for bacterial ribosomes, thus delaying both, peptide bond formation and peptidyl-tRNA hydrolysis. While BlaS does not inhibit stop codon recognition by the eukaryotic release factor 1 (eRF1), it interferes with eRF1’s accommodation into the peptidyl transferase center and subsequent peptide release. In human cells, BlaS inhibits nonsense-mediated mRNA decay and, at subinhibitory concentrations, modulates translation dynamics at premature termination codons leading to enhanced protein production.

## INTRODUCTION

Ribosome-targeting antibiotics are priceless tools in biochemistry and structural biology to dissect individual steps of translation and probe the modes of action of these antibiotics as well as of the factors involved in translation. A plethora of such compounds target translation initiation and elongation, and many of the antibiotics compromising elongation have been implemented in clinical practice (1). However, only very few antibiotics interfere with translation termination (1,2). Blasticidin S (BlaS) is an inhibitor of translation termination in bacteria (3). As early as in the 1960s, BlaS was known to inhibit protein synthesis in all kingdoms of life (3–7). BlaS is a peptidyl-nucleoside antibiotic composed of a cytosine, a pyranose sugar ring, and an N*-*methyl-guanidine tail. Structures show that BlaS binds to the P-site loop formed by ribosomal RNA within the peptidyl transferase center in the large ribosomal subunit of *Thermus thermophilus* (3), the archaea *Haloarcula marismorui* (8) and *Saccharomyces cerevisiae* (9). In contrast, other antibiotics known to interfere with peptide bond formation, such as puromycin or anisomycin, bind to the A-site in the peptidyl transferase center and inhibit aminoacyl-tRNA binding in archaea and yeast (8,9).

Structures of bacterial translating ribosomes showed that ribosome-bound BlaS displaces and deforms the 3′CCA tail of the P-site tRNA and distorts release factor 1 (RF1) binding in the A-site of the peptidyl transferase center, thereby preventing peptidyl-tRNA hydrolysis (3,10). BlaS also interferes with aminoacyl-tRNA binding to the ribosomal A-site and subsequent peptidyl transfer. However, peptide release is inhibited at considerably lower concentrations than peptide bond formation, and thus BlaS preferentially inhibits translation termination in bacteria (3,10). Since in *S. cerevisiae* BlaS binding to the P-site loop in the peptidyl transferase center was found to be conserved, inhibition of translation termination by BlaS was suggested to be conserved in eukaryotes and in bacteria (9).

In eukaryotic cells, translation termination is monitored by nonsense-mediated mRNA decay (NMD), a conserved eukaryotic mRNA surveillance pathway that targets mRNAs with premature stop codons for degradation (11–13). Active translation is required to recognize such nonsense mRNAs. Consequently, inhibition of translation by antibiotics cycloheximide, anisomycin or puromycin protects nonsense mRNAs from NMD (14–16). How NMD factors recognize nonsense mRNAs and differentiate between proper and improper termination remains enigmatic. According to current NMD models, translation termination at a premature termination codon is slow and inefficient compared to termination at a normal stop codon (11–13,17). This has been suggested to be caused by ribosome stalling at a premature stop codon, possibly due to inappropriate spacing between the stop codon and the termination-promoting poly(A)-binding protein in the 3′-untranslated region (18,19). Furthermore, the conserved NMD factor UPF3B has been shown to delay translation termination *in vitro* in a fully reconstituted human translation system (20).

Nonsense mutations constitute ∼20% of all human disease-associated single-base pair mutations (21). In this context, compounds that specifically modulate translation termination enabling enrichment of terminating ribosomes are urgently needed to better understand the difference between termination at normal versus premature termination codons and to design new treatment strategies for diseases caused by nonsense mutations.

Here, we dissected the impact of BlaS on mammalian translation, termination and on NMD. Using mammalian *in vitro* translation systems, we show that BlaS inhibits both translation elongation and termination. During termination, BlaS impairs peptide release and subsequent ribosomal dissociation by UPF3B. Cryogenic electron microscopy (cryo-EM) of BlaS-bound mammalian termination complexes reveals that, in contrast to bacterial complexes, accommodation of eRF1 in the peptidyl transferase center is inhibited in the mammalian ribosome due to a substantially larger deformation of the 3′CCA tail of the P-site tRNA, which is also predicted to interfere with peptide bond formation. In HeLa cells, BlaS treatment does not promote NMD by stalling termination, but instead stabilizes the premature stop codon-mutated mRNA. At low, sub-inhibitory concentrations BlaS increases production of the truncated nonsense protein while virtually not affecting global translation.

## MATERIALS AND METHODS

### *In vitro* transcription and capping

The C_*Firefly* LUC plasmid (22) encoding the *Firefly* luciferase gene was linearized by *Not*1 and transcribed *in vitro* using the T3 MEGAscript polymerase kit (Invitrogen, #AM1338) following the manufacturer's protocol. The mRNA was purified by LiCl precipitation, and subsequently capped using vaccinia capping enzyme (VCE) and *S*-adenosyl methionine (SAM, New England Biolabs, #B9003S). Briefly, 10 μg of mRNA was heated to 65°C for 10 min, then transferred to ice. The capping reaction was started in reaction buffer (50 mM Tris–HCl pH 8, 5 mM KCl, 1 mM MgCl_2_, 1 mM DTT) supplemented with 10 mM GTP and 2 mM SAM (final concentrations) with 1.6 μg VCE, incubated at 37°C for one hour, and subsequently the mRNA was purified by LiCl precipitation. The mRNA transcript encoding the 3xFLAG-tagged VHP protein was generated using the pSP64_3FLAG-VHPbeta68(TAG) plasmid as a DNA template as described previously (23). The DNA was linearized and amplified by PCR with forward primer (5′-TAATACGACTCACTATAGGGAATACAAGCTTGCTTGTTCTTTTTG-3′) annealing 100 bp upstream of the ORF, and reverse primer (5′-GAGGCGGTTTGCGTATTG-3′) annealing 200 bp downstream of the TAG stop codon. *In vitro* transcription and capping were performed as described above using T7 RNA polymerase (New England Biolabs, #M0251S) in the place of T3 MEGAscript following the manufacturer's protocol.

### Cloning of pSP64 3xFLAG-VHP-Sec61β _Y(TAG) plasmid

For peptide release assays, a 3xFLAG-tagged VHP protein with a C-terminal tyrosine residue before the stop codon, in place of the original valine, was created. The valine to tyrosine substitution was generated by PCR amplification of the pSP64 3xFLAG-VHP-Sec61β plasmid (23) to create the insert (forward primer: 5′-TAAAGATCATGACATCGATTACAAGGATGACGATG-3′, reverse primer: 5′- ACAGCTATGACATGATTACGAAGCTTCTAGTATTTGAGCCCAGGTGAATCTT-3′). The backbone was produced by digestion of pSP64 3xFLAG-VHP-Sec61β (TAG) with *Cla*1 and *EcoR*1, then Gibson ligation to assemble the final plasmid containing the tyrosine substitution (pSP64 3xFLAG-VHP-Sec61β (Tyr)(TAG)).

### Recombinant protein purification

Plasmids pProEx_Htb encoding eRF1 (20) and eRF1^AAQ^ (generated using a Q5 site-directed mutagenesis kit; New England Biolab #E0554S) were transformed into *Escherichia coli* BL21 Star (DE3), grown in 6 L of dYT until an OD_600 nm_ of 0.8, induced with 1 mM IPTG, and harvested after growth overnight at 20°C by centrifugation (Sorvall LYNX 6000 Superspeed Centrifuge (ThermoFisher) and Fiberlite™ F9-6 × 1000 LEX Fixed Angle Rotor (ThermoFisher), 5000 × g, 10 min, 4°C). Cell pellets were resuspended in 1× PBS supplemented with an additional 250 mM NaCl, 10 mM Imidazole, 1 mM DTT, two cOmplete EDTA-free protease inhibitor cocktail tablets (SigmaAldrich, #11873580001) and 1 mM PMSF. Cells were lysed using a French Press (Constant Systems, TS 0.75kV) operating at 25,000 PSI followed by centrifugation (Sorvall LYNX 6000 Superspeed Centrifuge (ThermoFisher) and Fiberlite™ F21-8 × 50y Fixed-Angle Rotor (ThermoFisher), 45 000 × g, 1 h, 4°C). The supernatant was loaded onto 1 ml HisTrap FF IMAC column (GE, #17531901). The column was washed with 25 column volumes (CV) and eRF1 eluted with a 50 CV gradient to 300 mM imidazole-containing lysis buffer. Eluted eRF1 was concentrated in lysis buffer lacking imidazole to a concentration of 300 μM, flash frozen, and stored at –80°C.

eRF3a was expressed in SF21 insect cells using the Multibac system (24) starting from the pFastBac_Htb plasmid as previously described (20). Cells were harvested by centrifugation (Sorvall LYNX 6000 Superspeed Centrifuge (ThermoFisher) and Fiberlite™ F9-6 × 1000 LEX Fixed Angle Rotor (ThermoFisher) 800 × g, 10 min, 4°C) and pellets were resuspended in lysis buffer (25 mM HEPES pH 7.4, 300 mM NaCl, 10 mM imidazole, 5% glycerol) and supplemented with a cOmplete EDTA-free protease inhibitor cocktail tablet before being lysed by sonication for 3 min on time at 70% amplitude using 5/10 s on/off cycling. The lysate was clarified by centrifugation at 45 000 × g (Sorvall LYNX 6000 Superspeed Centrifuge (ThermoFisher) and Fiberlite™ F21-8 × 50y Fixed-Angle Rotor (ThermoFisher) and supernatant incubated with 2 ml Ni-NTA superflow resin (Qiagen, #30410) for 1 h. The resin was then washed in lysis buffer containing 1 M NaCl, followed by 2 × 10 ml washes in lysis buffer and 4 × 10 ml elution steps with lysis buffer supplemented with 50, 100, 150 and 250 mM imidazole. Wash and elution fractions were analyzed by SDS-PAGE. eRF3a-containing fractions were diluted to 150 mM NaCl (final salt concentration) in buffer containing 25 mM HEPES pH 7.4, 1 mM DTT and loaded onto a 5 mL HiTrap Q XL column (GE, #17515901). Proteins were eluted over a 25 CV gradient from 150 to 1000 mM NaCl. Protein-containing fractions were analyzed by SDS PAGE and concentrated to 300 μM, flash frozen, and stored at –80°C. VCE was purified as previously described (25).

### *In vitro* translation luciferase assay

100 ng *Firefly* luciferase mRNA was added to rabbit reticulocyte lysate (RRL) (Green Hectares, Wisconsin, USA) in a reaction volume of 10 μl, as described previously (26). Blasticidin S (Sigma-Aldrich, #15205) was added simultaneously, and the *in vitro* translation reactions were incubated at 30°C for 30 min. Reactions were stopped by the addition of 30 μl of 1× lysis buffer from the luciferase assay kit (Promega, #E1500). The luciferase activity was detected following the manufacturer's protocol using a Synergy Neo2 plate reader (BioTek, Vermont, USA).

### Preparation of pre-termination complexes

*In vitro* translation reactions were conducted using an adapted RRL system (23,27). For biochemical assays, [^35^S]-labelled preTCs were prepared as follows: RRL *in vitro* translation reactions were supplemented with 2 μg of mRNA, 10 μM eRF1^AAQ^ and 0.1 mCi EasyTag™ [^35^S]-methionine (Perkin Elmer, #NEG772002MC) in a final volume of 200 μl for 20 min at 30°C. The reaction was stopped by adding 750 mM KOAc and 15 mM Mg(OAc)_2_. The preTCs were immobilized on 200 μl ANTI-FLAG M2 Affinity Gel (SigmaAldrich, #A2220) by incubation with gentle rotation at 4°C for 1.5 h, followed by washing 4X with wash buffer 1 (50 mM HEPES pH 7.4, 100 mM KOAc, 5 mM Mg(OAc)_2_, 0.1% Triton X-100, 1 mM DTT), 2× with wash buffer 2 (50 mM HEPES pH 7.4, 250 mM KOAc, 5 mM Mg(OAc)_2_, 0.5% Triton X-100, 1 mM DTT) and finally 4× with RNC buffer (50 mM HEPES, pH 7.4, 100 mM KOAc, 5 mM Mg(OAc)_2_, 1mM DTT). The preTCs were eluted with 0.1 mg/ml 3xFLAG peptide in RNC buffer, aliquoted, flash frozen and stored at –80°C.

### Peptide release assays

For peptide release assays, [^35^S]-labelled PreTCs were incubated with an equal volume of RRL and supplemented with the indicated amount of BlaS. The reactions were incubated at 30°C for 10 mins, then quenched by the addition of protein SDS-PAGE loading dye. For the peptide release time course assays, the reactions were supplemented with 800 nM BlaS and incubated at 30°C for the time points indicated. After quenching, the samples were separated on a 10% BIS:TRIS PAGE gel. All gels were dried and exposed to a phosphor screen and the amount of peptide measured using a Typhoon 9400 Variable Mode Imager (GE Healthcare/Amersham Biosciences) and images quantified using ImageJ (28).

### *In vitro* translation and toeprinting analysis of pre‐ and post‐termination complexes

**Figure 1.**
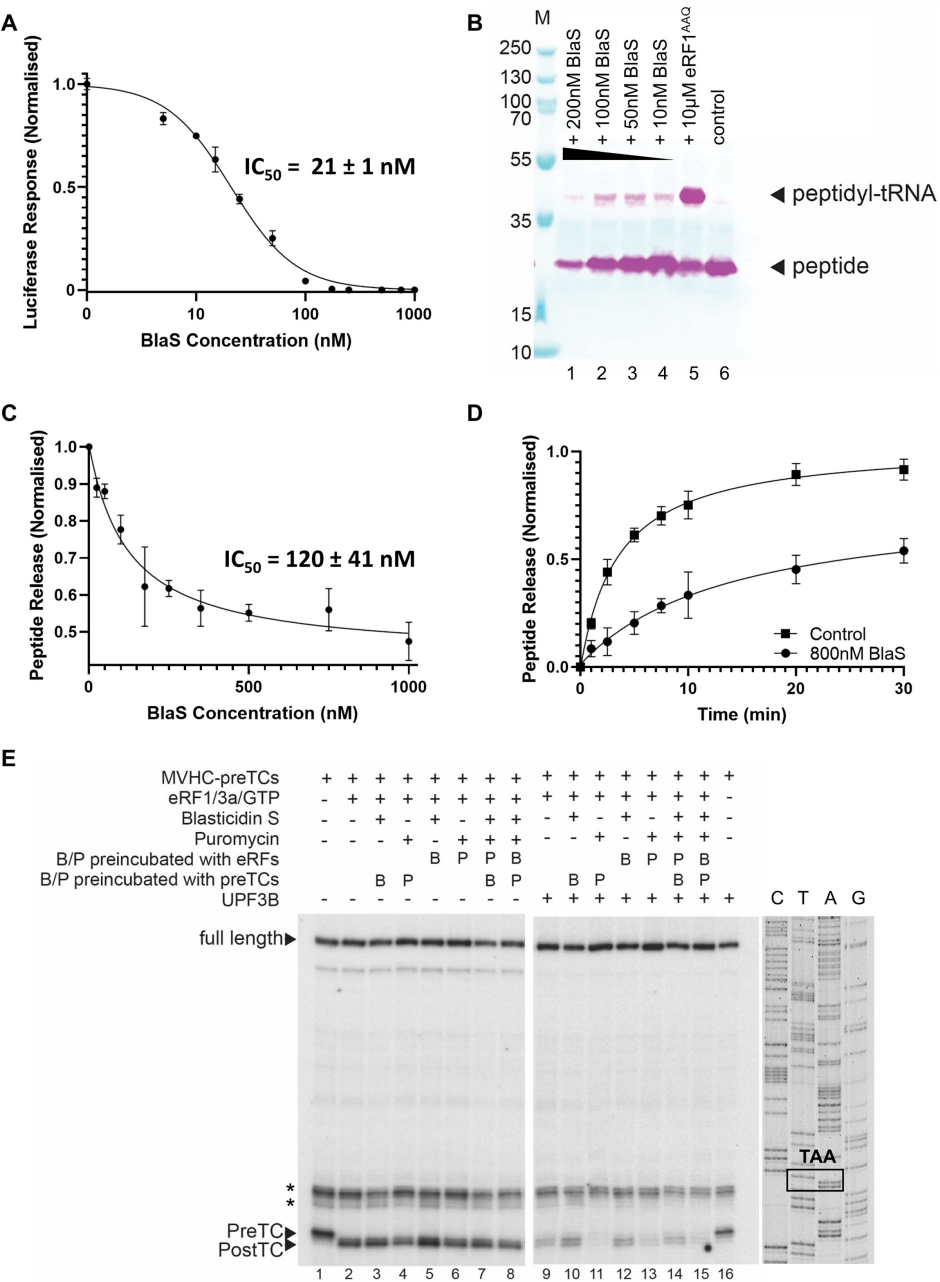
BlaS inhibitory effects on mammalian translation. (**A**) BlaS’ impact on *in vitro* translation determined via luciferase activity. Normalized response units are plotted against BlaS concentrations. (**B**) Immunoblot using anti-FLAG antibody to detect free peptide (lower band) and peptidyl-tRNA (upper band). Translation termination was inhibited by addition of 10μM eRF1^AAQ^ and different BlaS concentrations as indicated. (**C**) Peptide release of [^35^S]-methionine labelled 3xFLAG-Sec61β-VHP(Tyr) peptide from the ribosome in the presence of increasing concentrations of BlaS. Addition of BlaS decreases the ratio of free peptide compared to peptidyl-tRNA. (**D**) Time-dependence of peptide release inhibition by BlaS. The ratio of released peptide versus peptidyl-tRNA is determined in the absence (squares) and presence (dots) of 800 nM BlaS. (**E**) Left autoradiogram: Toeprinting analysis of ribosomal complexes obtained by incubating preTCs assembled on MVHC-stop mRNA (MVHC-preTCs) with eRF1, eRF3a, GTP and combinations of 5 μg/ml BlaS and 1 mM puromycin at 1 mM free Mg^2+^. The positions of preTCs, postTCs and full-length cDNA are indicated. Asterisks mark initiation and elongation complexes. Right: Toeprinting analysis of ribosomal complexes obtained by incubating preTCs with UPF3B, eRF1, eRF3a, GTP, and combinations of 5 μg/ml BlaS and 1 mM puromycin. Disappearance of the postTC band indicates dissociation of ribosomal complexes and concomitant release of mRNA, as indicated by more full-length cDNA. The gel on the left was exposed 2× longer than gel on the right. The slightly lower intensity of the postTC toe-print band generated after incubation with puromycin (lanes 4, 6, 8) is likely due to puromycin-treated preTCs being relatively unstable at the low Mg^2+^ concentrations used (63). Error bars indicate the standard deviation of three replicates.

*In vitro* termination and toeprinting analysis were performed as previously described (20). In the data displayed in Figure 1, the release factors or the preTCs were incubated for 5 min at 37°C with 5 μg/ml BlaS or 1 mM puromycin (with respect to the final volume of the termination reaction) and subsequently combined. Translation termination was performed in the presence or absence of UPF3B and allowed to proceed for 5 min.

### *In vivo* luciferase reporter assay

The Luciferase reporter system has been described elsewhere (29). Briefly, 1 × 10^5^ HeLa cells/well were seeded in six-well-plates and transfected after 24 h with 0.6 μg/well of the reporter plasmids pCI-*Renilla*-HBB WT or pCI-*Renilla*-HBB-Nonsense-Mutation 39 (NS39). 0.35 μg/well of the pCI-*Firefly*-luciferase plasmid and 0.2 μg/well of a YFP-expression vector were co-transfected for quantification and visual assessment of transfection efficiency. After 16 h, the cells were washed with full medium and treated for 3 h with BlaS or left untreated as indicated in Figure 5 and Supplementary Figure S5. Cells were lysed in 200 μl/well Passive Lysis Buffer (Promega #E1941). 3 μl were used to measure luciferase luminescence in the Centro LB 960 luminometer (Berthold Technologies, Germany) using the Dual-Luciferase Reporter Assay System (Promega # E1910). *Renilla* luciferase signals were normalized to the *Firefly* luciferase control signals and subsequently normalized WT and NS39 expression levels were compared. In indicated cases transfected cells were lysed in RIPA buffer. 10–15 μg of total protein was separated on 10% SDS-PAGE gels and immunoblotted. Expression of *Renilla*-HBB fusion proteins was monitored using an anti-*Renilla* antibody, and *Firefly* luciferase was detected using an anti-*Firefly* luciferase antibody (both Merck, Darmstadt, Germany).

### Quantitative real-time PCR

The remainder of above lysate was extracted with TRIzol (ThermoFisher #15596026) to isolate total mRNA. 2 μg total mRNA was used to generate cDNA. The quantitative RT-PCR was performed on a StepOnePlus™ machine (Applied Biosystems/ThermoFisher), using Absolute™ SYBR green mix (ThermoFisher #AB1158B). The primers used were: HBBex1/2sense: 5′CTGGGCAGGCTGCTGGTG3′; HBBex2/3 as: 5′CGTTGCCCAGGAGCCTGAAG3′; *Firefly* sense: 5′AGAGATACGCCCTGGTTCCT3′; *Firefly* antisense: 5′ATAAATAACGCGCCCAACAC3′. *Renilla*-HBB WT/NS39 expression levels were normalized to *Firefly* expression levels and subsequently the normalized *Renilla*-HBB-NS39 levels (with and without treatment) were compared to the respective WT levels.

### Sucrose cushion centrifugation and luciferase detection

A 1 ml 1 M sucrose cushion in RNC buffer was topped with 300 ul of prepared HeLa cell lysate transfected as above with pCI-*Renilla*-HBB-WT, pCI-*Renilla*-HBB-NS39, or pCI-*Firefly*-Luciferase mRNA generated from a 10 cm dish lysed in 20 mM Tris, pH 7.5, 100 mM KAc, 5 mM Mg(OAc)_2_, 0.5% NP-40 containing protease and RNase inhibitor. These cells were transfected and treated (for 3 h) with 5 or 100 ug/ml BlaS or left untreated and lysed as above. Ultracentrifugation was performed at 55 000 rpm for 3 h using a TLA-55 rotor and an Optima Ultracentrifuge (Beckman Coulter) at 4°C.The supernatant was removed, and the ribosomal pellet was dissolved in an equal volume RNC buffer. *Renilla* and *Firefly* luciferase detection was conducted as above using the manufacturers recommended protocol but using a Synergy Neo2 plate reader (BioTEK, USA).

### Statistical analysis

For statistical analysis SigmaPlot v13 (SyStat Software) was used. Results were considered statistically significant if *P* values were $ \le$ 0.05, with one star indicating 0.01 < *P*$ \le$ 0.05, two stars for 0.001 < *P*$ \le$ 0.01, and three stars for *P*$ \le$ 0.001. Briefly, one-way ANOVAs were applied for multiple comparisons (two-tailed), followed by a multiple comparison analysis. The statistical analysis was based on at least three replicates with degrees of freedom and normality tests detailed in the respective figure legends.

### Cryo-EM sample preparation

PreTCs samples for cryo-EM were prepared as above with the following modifications: The *in vitro* translation reaction volume was increased to 2 ml and supplemented with 20 μg capped mRNA and methionine at a final concentration of 50 μM. The reaction was incubated at 32°C for 25 min. The preTCs were incubated for 3 h with ANTI-FLAG M2 Affinity Gel, followed by washing, and eluting twice in 200 μl incubations of 0.1 mg/ml 3xFLAG tag peptide in storage buffer at room temp for 25 min. Eluted ribosomes were isolated via 0.5 M sucrose cushion ultracentrifugation (Beckman Coulter TLA-55 rotor, 55 000 rpm, 3 h, 4°C). The ribosomal pellets were washed with 100 μl storage buffer before being resuspended in 100 μl storage buffer by agitation at 900 rpm at 4°C. Ribosome concentrations were determined using the Nanodrop (ThermoFisher, #ND-ONEC-W) and supplemented with 5× molar excess eRF1^AAQ^ and eRF3a with 1 mM GTP and 5× molar excess BlaS followed by ultracentrifugation (Beckman Coulter TLA-120.2 rotor, 100 000 × g, 1 h, 4°C). The pelleted ribosomal complexes were resuspended in 50 μl storage buffer and resuspended by agitation at 900 rpm at 4°C. Ribosomal concentration as measured and then supplemented with 5× molar excess eRF1^AAQ^ and eRF3a with 1 mM GTP and 5× molar excess BlaS (final) prior to grid preparation.

Cryo-EM grids were prepared using 165 nM ribosomal complexes by applying 3 μl sample at 15°C and 70% relative humidity to glow discharged R2/2 quantifoil grids (Electron Microscopy Sciences, #Q3100CR2) covered with a ∼40 Å thick layer of amorphous carbon prepared using a carbon coater (Leica EM ACE 600. Using a Leica EM GP2 plunge freezer, the sample was incubated 30 s, blotted for 1.1 s, and vitrified in liquid ethane maintained at liquid nitrogen temperature. Grids were stored in liquid nitrogen until data collection.

### Data collection and image processing

Data were collected with a FEI Talos Artica TEM using quasi-automated collection software (EPU) equipped with a 200 kV X-FEG electron source. Movies were recorded on a K2 Summit detector (Gatan) at super-resolution mode, at a dose rate of 5.24 e^−^/Å^2^/s with a total exposure time of 8 s, for an accumulated dose of 41.9 e^−^/Å^2^. Intermediate frames were recorded every 0.2 s with a total number of 40 movie frames per micrograph. A calibrated magnification of 100 000× was used yielding a physical pixel size of 1.35 Å (super-resolution pixel size is of 0.675 Å). Defocus values ranged from −0.4 to −2.0 μm with a step size of 0.4 μm (Supplementary Table S1). Movie frames were first aligned using whole-image motion correction (30) for reduction of beam-induced image blurring. Micrographs with indications of poor contrast, astigmatism, charging, or contamination were discarded. Defocus values of the aligned micrographs were estimated using CTFFIND4 (31). Further, micrographs with estimated resolutions above 5 Å were discarded. Relion 3.0 (32) was used to manually pick particles (∼3,500) and generate initial reference-free 2D averages. This was followed by automated-picking using Relion 3.0 (32) which yielded a total of 730,463 particles. Particle images were extracted with a box size of 600 (binned to 300) yielding a pixel size of 1.35 Å. 2D class averages were generated and subsequently used to further discard poor particles or non-ribosomal particles yielding 295,840 particles. An initial 3D model was generated using 50,000 particle images using Relion 3.0 (32). This model was used for further 3D classifications into eight classes (angular sampling of 7.5° for 5 pixels with local searching over 25 iterations) (Supplementary Figure S2). The five best classes were pooled (255,549 particles) and subjected to a second round of 3D classification into eight classes (angular sampling of 3.5° for 3 pixels with local searching over 25 iterations) and refinement. We identified three 3D classes with ribosome bound to BlaS with (i) an empty A-site (Empty-A), (ii) eRF1 bound A-site (eRF-Bound) and (iii) adopting hybrid P/E and A/P tRNA state (Hybrid) comprising 103,842, 18,397 and 29,879 particles respectively (Supplementary Figure S2). Bayesian particle polishing and contrast transfer function (CTF) refinement (33) in Relion 3.0 (32) resulted in a resolution of 3.13 Å for the Empty-A map (Fourier shell correlation (FSC) cut-off 0.143), a resolution of 4.1 Å for the eRF-Bound map, and 3.82 Å for Hybrid map. A B-factor of −50 was used for map sharpening (the B*-*factor value was determined empirically to obtain a well-interpretable density map) (Supplementary Table S1). Local resolution was determined using Relion 3.0 (32).

### Model construction and refinement

For the empty A-site model, an 80S ribosome (PDBID: 3JAH (23), without the chains for eRF1^AAQ^ and ABCE1) was docked into the map of the empty A-site map using Chimera (34). BlaS was modelled using constraints taken from the 70S ribosome structure with BlaS (PDBID: 4V9Q (3)), docked into density corresponding to BlaS (using constraints taken from ELBO in Phenix using code BLS) using COOT’s ligand fit tool (35,36). Iterative real space refinements against the amplitudes and phases from the cryo-EM experimental map (which remained unchanged during the refinement) were carried out using Coot (0.8.9.2EL (35)) and Phenix (1.17.1–3660 (36)) using blurred maps (B-factors between –25 and –50). The same processing steps were utilized for the hybrid A/P-P/E, utilizing PDBID 6HCJ (37) (without poly(A) nascent chain and mRNA) as a starting model. For the eRF1/eRF3a-bound model PDBID 5LZT (27) was used as a starting model. The quality of the models was scrutinized using MolProbity (38) (Supplementary Table S1).

## RESULTS

### Blasticidin S impact on mammalian translation

BlaS inhibits translation in prokaryotic and eukaryotic cells and specifically impairs translation termination in bacteria (3–5). However, detailed biochemical and biophysical studies in a mammalian system are lacking. Here, we used an adapted rabbit reticulocyte lysate (RRL) translation system for *in vitro* translation (23) to elucidate the effect of BlaS on eukaryotic translation termination. First, we determined the steady state efficiency of translation using a luciferase reporter assay in the presence of different BlaS concentrations. Capped *Firefly* luciferase mRNA was added to RRL (23), and synthesis of the luciferase enzyme was monitored in a high-throughput plate reader by measuring Luciferin substrate turnover. Upon titration of BlaS into the translation reaction, the synthesis of luciferase decreased in a dose-dependent manner (Figure 1A). Inhibition of translation was observed at BlaS concentrations as low as 5 nM, and translation was completely inhibited by concentrations above 175 nM. The half-maximal inhibitory concentration (IC_50_) was determined to be 21 nM.

Although our luciferase assay confirmed the overall inhibition of mammalian translation by BlaS, it did not inform at which stage translation is affected. To distinguish between translation elongation and termination, we used an mRNA encoding an N-terminally 3xFLAG-tagged fusion protein consisting of truncated Sec61β furnished with an autonomously folding 15 kDa villin head piece (VHP) domain (23) for *in vitro* translation. Translation of the 3xFLAG-Sec61β-VHP mRNA in RRL produces a single product that can be detected by Western blot using an anti-FLAG antibody (Figure 1B, lane 6). Addition of an excess (10 μM) of an inactive eRF1 mutant (eRF1^AAQ^) inhibits peptidyl-tRNA hydrolysis by wildtype eRF1, leading to a ribosomal complex stalled at the stop codon with peptidyl-tRNA in the P-site and eRF1^AAQ^ in the A-site. Accordingly, Western blot analysis of RRL translation reactions in the presence of eRF1^AAQ^ revealed, in addition to the band corresponding to the free 3xFLAG-Sec61β-VHP protein, a strong band with slower electrophoretic mobility corresponding to unhydrolyzed peptidyl-tRNA (Figure 1B, lane 5). Translation in the presence of 10–200 nM BlaS also produced peptidyl-tRNA bands, but of significantly lower intensity compared to translation in the presence of eRF1^AAQ^ (Figure 1B, compare lanes 1–4 with lane 5). With increasing BlaS concentrations, both the bands corresponding to the free and the t-RNA-bound protein decrease in intensity (Figure 1B, lanes 1–4). The generation of the peptidyl-tRNA already at low BlaS concentrations and the decrease of both the free and tRNA-bound protein at higher BlaS concentrations indicate that *in vitro*, BlaS inhibits mammalian translation both at the elongation and the termination stage.

### BlaS inhibits peptide release from the ribosome

Previous studies using bacterial ribosomes have shown that BlaS inhibits peptide release (3,10). To further investigate BlaS’ mode of action in eukaryotes, we performed peptide release assays using pre-termination complexes (preTCs) purified from RRL. Because tyrosine has been shown to be more efficiently released than valine by release factors in bacteria (39) we replaced the valine codon in the penultimate position before the UAG stop codon with a tyrosine codon. PreTCs were prepared using this modified 3xFLAG-Sec61β-VHP mRNA (3xFLAG-Sec61β-VHP(Tyr)). *In vitro* translation was performed in the presence of [^35^S]-methionine and eRF1^AAQ^. Affinity purification via the N-terminal 3xFLAG tag included a high-salt wash step to remove all release factors from the preTCs. To investigate peptide release in the presence of increasing concentrations of BlaS, wild-type eRFs and GTP were added to these preTCs (non-limiting concentrations) and termination proceeded for 10 minutes. After SDS PAGE and autoradiography, peptidyl-tRNA and free peptide bands were quantified (Supplementary Figure S1). As expected, peptide release decreased with increasing BlaS concentrations (Figure 1C; Supplementary Figure S1A). The IC_50_ for peptide release was determined to be 120 nM and thus considerably higher than the IC_50_ determined for translation inhibition (21 nM, Figure 1A) indicating that inhibition of termination is only partially responsible for BlaS’ effect on translation.

Next, we explored the time-dependence of peptide release by BlaS. In the presence of eRFs, but not BlaS (control reaction), half-maximum peptide release from the preTCs was achieved within ∼4 minutes. When the preTCs were pre-incubated with 800 nM BlaS before the addition of eRFs, peptide release was substantially inhibited, and half-maximal release was achieved only after 25 minutes (Figure 1D, Supplementary Figure S1B).

We could not exclude that BlaS inhibits a step prior to peptide release. Therefore, we tested if BlaS also interferes with stop codon recognition by eRF1. Using preTCs that contain a MVHC-tetrapeptide-tRNA generated in a human reconstituted translation system (20,40), we performed primer extension inhibition assays (toeprinting assays). PreTCs with peptidyl‐tRNA^Cys^ in the ribosomal P‐site and the stop codon in the A site generate a toe-print band 16 nucleotides 3′ to the U of the P-site UGC (Cys) codon. Stop codon recognition by eRF1^WT^ or eRF1^AGQ^ is detected as a +1 or +2 nucleotide shift from a preTC to a post-termination complex (postTC) (Figure 1E, lanes 1, 2, Supplementary Figure S1C) (40). The formation of postTCs, i.e. the recognition of the stop codon in the ribosomal decoding center by eRF1, was not impaired by the presence of BlaS (Figure 1E, lanes 3, 5, 7, 8) and/or by the presence of puromycin, which causes peptide release (Figure 1E, left gel, lanes 4, 6, 7, 8). This finding was independent of the order of addition of BlaS to the preTCs (Figure 1E, lanes 3, 7), or to the eRFs (Figure 1E, lanes 5, 8). Thus, BlaS does not interfere with stop codon recognition by eRF1.

We previously reported that the NMD factor UPF3B dissociates postTCs after peptide release by eRFs in a manner reminiscent of ribosome recycling by eIF1, eIF1A, eIF3 and eIF3j (20,41). Ribosome dissociation is evidenced by the weakening of postTC toeprints and increased intensity of the full-length mRNA band (Figure 1E, lane 9). When peptide release is further enhanced by puromycin, UPF3B causes a disappearance of postTC toeprints indicating complete ribosome dissociation (Figure 1E, lanes 11, 13, 15). In contrast, BlaS interferes with postTC dissociation by UPF3B and preserves postTC toeprints, similar to the eRF1^AGQ^ mutant (Figure 1E, lanes 10,12,14, Supplementary Figure S1C, compare lanes 5, 6, 8 and 10). This agrees with our finding that BlaS inhibits peptide release (Figure 1C,D), and further confirms our earlier finding that UPF3B triggers ribosome dissociation after peptidyl-tRNA hydrolysis. It indicates that the efficiency of UPF3B in ribosome dissociation directly reflects the completeness of peptide release by puromycin (Figure 1E, lane 11) or the eRFs (Figure 1E, lane 9) (20). Interestingly, in the toeprinting experiments, pre-binding of BlaS to the preTCs interferes to some extent with peptide release by puromycin and subsequent dissociation of ribosomes by UPF3B (Figure 1E, comparing lanes 15 and 14). This may be due to a competition of the two antibiotics for a similar binding site reminiscent of the one described in *Escherichia coli* (42,43), or reflect a distortion of the peptidyl transferase center by BlaS interfering with peptide release by puromycin. In conclusion, our *in vitro* termination experiments confirm that BlaS does not interfere with stop codon recognition by eRF1 but prevents efficient peptidyl-tRNA hydrolysis by the eukaryotic release factors and subsequent ribosome dissociation by UPF3B.

### Cryo-EM of mammalian ribosomal termination complexes with BlaS

**Figure 2.**
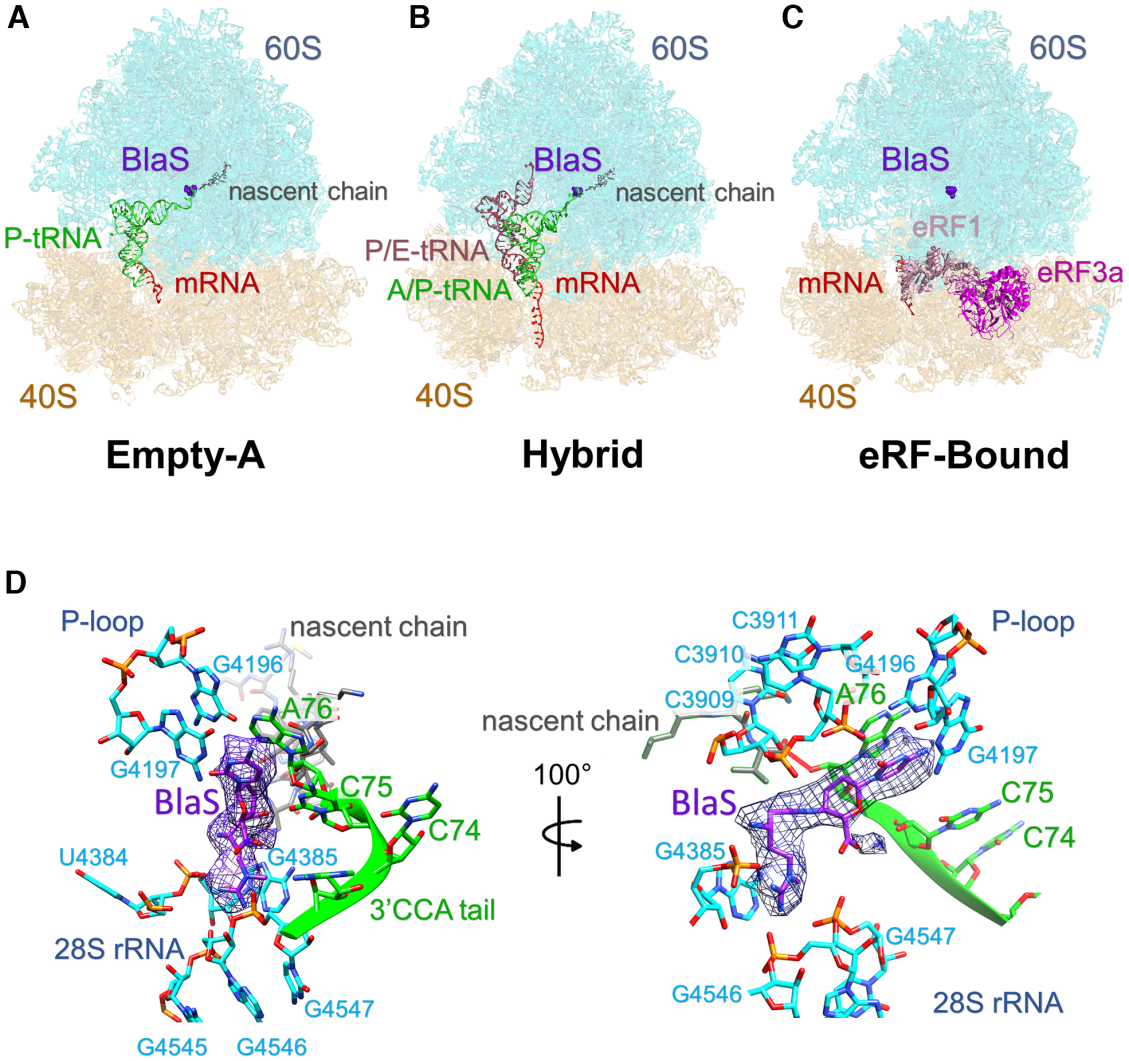
Cryo-EM structures of mammalian termination complexes (TCs) with BlaS. Three major classes were identified in our data. (**A**) TC-structure with empty A-site and with BlaS and peptidyl-tRNA bound to the P-site (Empty-A, 3.1 Å resolution) representing ∼35% of the particles. (**B**) Structure with BlaS, empty tRNA in the P/E hybrid state and peptidyl-tRNA in the A/P hybrid state (Hybrid, 3.8 Å resolution) representing ∼11% of the particles. (**C**) TC-structure with BlaS in the peptidyl transferase center and eRF1/eRF3a in a pre-accommodation state bound to the decoding center of 40S (eRF-Bound, 4.1 Å resolution) representing ∼6% of the particles. In panels A–C, the 60S subunit is depicted in cyan, the 40S in orange, mRNA in red, nascent chain in grey, peptidyl-tRNA in green, empty tRNA in light red, eRF1 in pink, eRF3a in magenta, and BlaS in purple. (**D**) Close-up view into the peptidyl transferase center in the Empty-A structure. EM density (purple mesh) corresponding to BlaS (purple) bound to 28S rRNA (cyan) and by the 3′ CCA tail of the P-site peptidyl-tRNA (green, nascent chain grey) is shown. Left: same view as in panel A; right: 100° rotated about the Y-axis.

**Figure 3.**
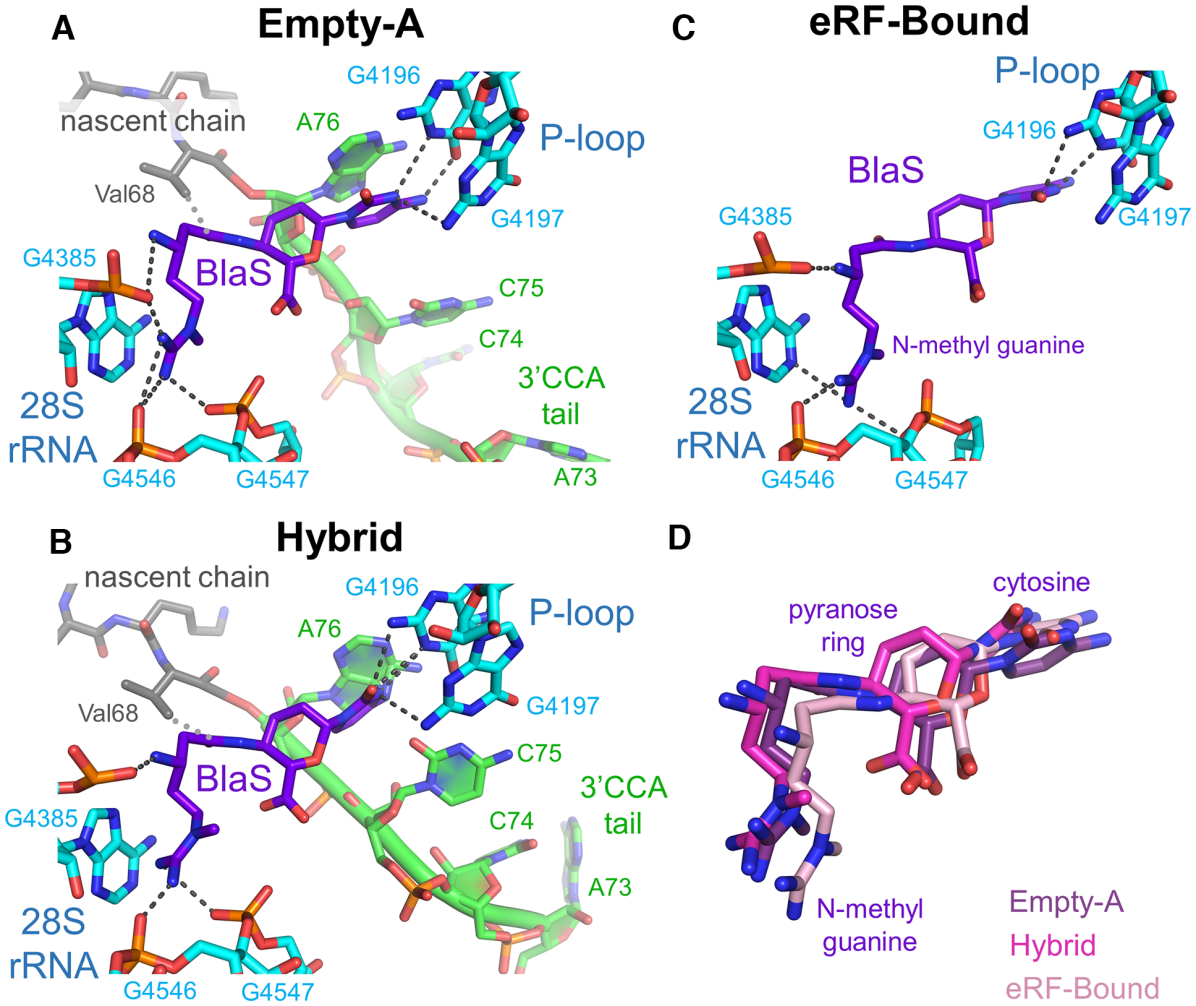
BlaS binding to the P-site in the 60S peptidyl transferase center. Contacts formed by BlaS (purple) with 28S rRNA bases in the P-site (cyan) and with the 3′CCA tail of the bound peptidyl-tRNA (green and grey for nascent chain) in the Empty-A structure (**A**), the Hybrid structure (**B**) and the eRF-Bound structure (**C**). Hydrogen bonds are shown by black dashed lines, van der Waals contacts are shown by grey dotted lines. (**D**) The relative positions and orientations of bound BlaS in these three structures are overlaid with BlaS colored magenta in the Empty-A structure, pink in the Hybrid structure, and light pink in the eRF-Bound structure.

In bacteria, archaea, and yeast, BlaS binding occurs in the P-site of the peptidyl transferase center of the large ribosomal subunit (3,8–10). In bacterial ribosomes, BlaS binding causes a distortion of the 3′CCA tail of the P-site peptidyl-tRNA pushing this region outward in the direction of the A-site (3,10). Despite this distortion of the P-site tRNA, BlaS does not prevent accommodation of RF1 into the 50S A-site in bacteria (10). To understand BlaS’ impact on mammalian translation termination, we solved the cryo-EM structure of ribosomal complexes with BlaS, eRF1, and eRF3a in the presence of GTP (Figure 2). PreTCs were generated using the RRL *in vitro* translation system and mRNA encoding 3xFLAG-Sec61β-VHP(Val) described above (23). eRF1^AAQ^ was added in excess to the translation reaction to trap termination complexes, followed by FLAG affinity purification and extensive wash steps to liberate any bound factors including eRF1^AAQ^ from the ribosome. These purified, release factor-free preTCs were incubated with 10 μM BlaS and 5× molar excess of eRF1, eRF3a and 1 mM GTP and then used for cryo-grid preparation and EM data collection (Supplementary Figure S2, Supplementary Table S1). 3D classification yielded three major classes: In class 1, called ‘Empty-A’ (103,842 particles, 3.1 Å resolution), the ribosomal P-site is occupied by peptidyl-tRNA, and the A-site is free of any bound eRFs (Figure 2A, Supplementary Figures S2, S3A, Supplementary Table S1). In class 2, called ‘Hybrid’ (29,879 particles, 3.8 Å resolution), tRNAs are bound in hybrid P/E and A/P states and no eRFs are bound (Figure 2B, Supplementary Figures S2, S3B, Supplementary Table S1). In class 3, called ‘eRF-Bound’ (18,937 particles, 4.1 Å resolution), the P-site tRNA is present but poorly resolved (Supplementary Figure S4C), and therefore the P-site tRNA in this structure is not modelled. The A-site in the small subunit (the decoding center) is bound by eRF1, and eRF1 and eRF3a are in the pre-accommodation state (Figure 2C, Supplementary Figures S2, S3C, Supplementary Table S1) (27). All three structures have BlaS bound in the 60S peptidyl transferase center (Figure 3, Supplementary Figure S4).

### BlaS binding distorts P-site tRNA and prevents eRF1 accommodation

**Figure 4.**
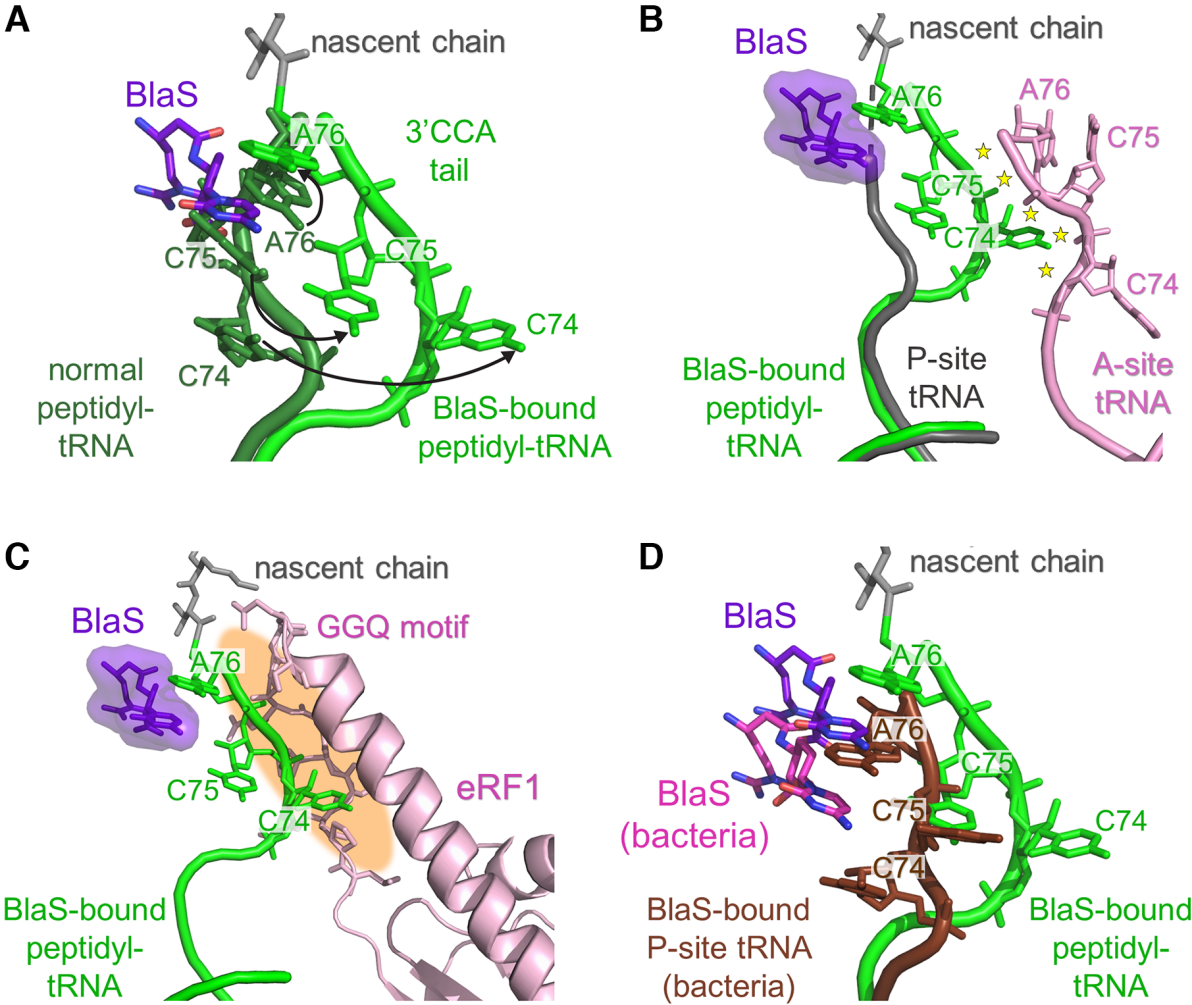
Mis-positioning of the peptidyl-tRNA 3′CCA tail in the presence of BlaS. (**A**) Overlay of BlaS-bound P-site tRNA in the Empty-A structure (light green, BlaS purple) and P-site tRNA in a normal (not BlaS-bound) mammalian ribosomal termination complex (dark green) (PDBID: 3JAH, (23)). Arrows indicate displacement of the 3′CCA bases. (**B**) Overlay of BlaS-bound P-site tRNA in the Empty-A structure (light green, BlaS purple) and of an elongating *Thermus thermophilus* 70S ribosomal complex with acylated A-site (pink) and P-site tRNAs (grey) (PDBID: 4V5D, (44)). Minor steric clashes are indicated by yellow stars. (**C**) Comparison of BlaS-bound P-site tRNA in the Empty-A structure (light green, BlaS purple) with the structure of the mammalian termination complex with eRF1 accommodated in the A-site (PDBID: 5LZU, (27)). The P-site tRNA in the termination complex was omitted for clarity. Severe steric clashes are indicated in orange. (**D**) Comparison of BlaS-bound bound P-site tRNA in the Empty-A structure (light green, BlaS magenta) with the crystal structure of *Th. thermophilus* 70S ribosome-tRNA complex (dark red) bound to Blas (pink) (PDBID: 4V9Q, (3)) showing differences in BlaS binding to bacterial and eukaryotic ribosomal complexes. These result in larger distortion of the tRNA 3′CCA tail in the mammalian complex. The structures were aligned for the 28S (23S in panel D) rRNA residues (not shown for clarity).

In the Empty-A cryo-EM structure representing ∼35% ribosomes, density for BlaS is observed near the 3′CCA tail of the P-site peptidyl-tRNA (Figure 2D). Hydrogen bonds are formed between the cytosine part of BlaS and 28S rRNA bases G4196 and G4197 of the P-loop, but we do not observe Watson-Crick base pairing as in bacteria and archaea (3,8). The N-methyl-guanidine tail of BlaS interacts with the phosphate backbone of G4385, G4546 and G4547 (Figure 3). The P-site peptidyl-tRNA forms a base-stacking interaction with the cytosine part of BlaS via residue A76 of the 3′CCA tail. This leads to displacement of C74 and C75 and distortion of the 3′CCA end (Figures 2D and 3A). Interestingly, we observe a van der Waals interaction between BlaS and the sidechain of the terminal valine (Val68) of the nascent chain which further contributes to BlaS coordination and to distortion of the peptidyl-tRNA (Figure 3A). Overall, BlaS binding to the P-site causes displacements of up to 9.5 Å for backbone phosphates and up to 14.5 Å for the bases of the 3′CCA tail of the tRNA relative to their normal position in the P-site of the peptidyl transferase center (Figure 4A) (23). Beyond the 3′CCA tail, the binding of BlaS has virtually no impact on the tRNA conformation or the architecture of the 40S decoding center. In the Hybrid and eRF-Bound cryo-EM structures, BlaS density is observed in the P-site of the 60S subunit at the same position (Figure 3B,C,D). However, small differences are found in the positioning of BlaS’ cytosine and *N*-methyl-guanidine tail resulting in a slightly altered hydrogen bonding network and clearer base stacking with both C75 and A76 of the 3′CCA tail (Figure 3B, C).

An overlay of A-site and P-site tRNAs from a mammalian ribosomal elongation complex (44), with BlaS and peptidyl-tRNA in the Empty-A structure reveals that the distorted P-site tRNA would cause clashes with the A-site tRNA (stars in Figure 4B). Accordingly, accommodated A-site aminoacyl-tRNA would have to adopt a different, sub-optimal conformation in the BlaS-bound ribosome, explaining how BlaS interferes with peptide bond formation and thus elongation.

Structures of bacterial RFs bound to ribosomal complexes have all indicated a strong propensity of RFs to exist in the accommodated state (45–48). To determine a cryo-EM structure of the pre-accommodation state of the bacterial termination complex, a special hyper-accurate RF1 variant combined with termination step inhibition by incubation with BlaS was required (49). Eukaryotic termination complexes show eRF1 in the accommodated state (23,27,50) and, in the presence of non-hydrolysable GTP analogues, in the pre-accommodated state (27,51). Our three cryo-EM structures all show an empty 60S A-site despite efficient stop codon recognition by eRF1 in toeprinting assays (Figures 1E and 2). This was unexpected because we added excess GTP to our sample and thus stop codon recognition should lead to activation of eRF3a, GTP hydrolysis, and accommodation of eRF1 into the A-site of the peptidyl transferase center. However, density for eRF1 was found only in the decoding center of 40S in one of the structures (Figure 2C). This eRF-bound structure, which presents ∼6% of the particles, is lower resolution (4.1 Å) compared to the other two structures, and the local resolution of eRF1 and eRF3a is even lower (ca 4.5–6.0 Å), suggestive of a degree of dynamic conformational sampling within the eRF1–eRF3a complex (Supplementary Figure S3C). This likely reflects attempted accommodation by eRF1 which is being prevented by BlaS binding. An overlay of a previous mammalian ribosomal termination complex with accommodated eRF1 (27) with our Empty-A structure reveals a steric clash between the distorted 3′CCA tail of the BlaS-bound peptidyl-tRNA and the M domain of eRF1, including the catalytic GGQ motif of eRF1 responsible for peptide release (Figure 4C). In contrast, a structure of a bacterial 70S termination complex bound to RF1 and BlaS showed accommodation of RF1 (10), but with distortion of the catalytic GGQ motif of RF1. This difference is most likely due to a substantially larger distortion of the mammalian peptidyl-tRNA in the presence of BlaS compared to bacterial tRNA in the P-site (3) (Figure 4D). BlaS is localized further towards the 3′ end of the tRNA in the mammalian complex, closer to the site of the nucleophilic attack, potentially explaining the observed larger shift in the 3′CCA tail. This difference in distortion of the P-site tRNA may allow accommodation of bacterial RF1 but not of eRF1. Therefore, the absence of eRF1 accommodation in our structures is likely explained by this comparatively large distortion of the 3′CCA tail of peptidyl-tRNA. Consequently, the steric clash in this case is much more severe, interfering with eRF1 accommodation into the peptidyl transferase center in the presence of BlaS and subsequent peptide release.

### Sub-inhibitory BlaS concentrations moderately stabilize nonsense mRNA and induce production of a truncated protein

**Figure 5.**
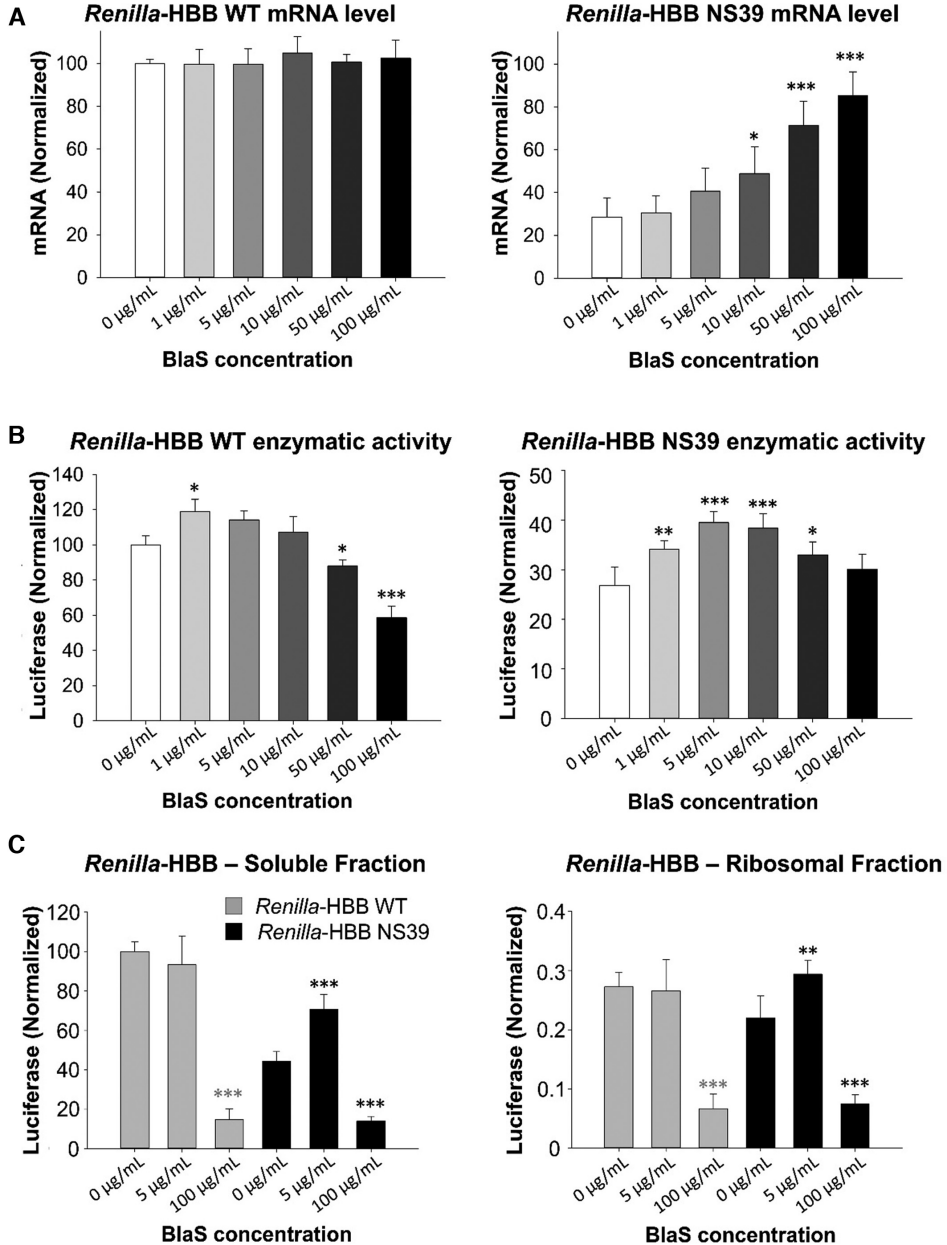
Quantification of *Renilla*-HBB reporter mRNA levels and associated luciferase activity in transfected HeLa cells after incubation with different concentrations of BlaS. (**A**) qRT-PCR analysis of the *Renilla*-HBB wildtype reporter mRNA (left) and of the *Renilla* HBB NS39 reporter mRNA (right), following treatment with indicated BlaS concentrations. The levels of reporter mRNA are shown as percentage of *Renilla*-HBB WT mRNA not treated with BlaS (0 μg/ml BlaS), with the SD of three or more independent experiments. Co-expressed *Firefly* luciferase mRNA levels were used to normalize the levels of*Renilla*-HBB mRNA. (**B**) Reporter luciferase activity following treatment with indicated BlaS concentrations, normalized to wildtype *Renilla* luciferase-HBB activity not treated with BlaS (0 μg/ml BlaS), with the SD of three or more independent experiments. (**C**) Reporter luciferase activity following treatment of transfected cells with two indicated concentrations of BlaS and sucrose cushion centrifugation. Left: supernatant fraction, right: ribosomal pellet fraction. Luciferase activity of the *Renilla*-HBB WT reporter is shown in grey; luciferase activity of the *Renilla*-HBB NS39 reporter in black. The activity of *Renilla*-HBB reporter protein is normalized to *Renilla*-HBB WT protein sample not treated with BlaS (0 μg/ml BlaS), with the SD of three or more independent experiments. One-way ANOVA’s (Holm-Šidák) statistical significance tests (α = 0.05) are indicated with asterisks identifying those with a *P* value 0.01 < *P*$ \le$ 0.05 having one, 0.001 < *P*$ \le$ 0.01 having two, and three for *P*$ \le$ 0.001. Panel A denoting mRNA and panel B for associated luciferase measurements had 3 measurements per triplicate resulting in degrees of freedom (DF) of 47. Panel B denoting luciferase measurements from sucrose cushions had 7 measurements per triplicate yielding DF = 62. Normality of distributions was assessed via Shapiro Wilk tests for each panel with *P* = 0.058 and *P* = 0.180, *P* = 0.671 and *P* = 0.783, *P* = 0.073 and *P* = 0.077 for mRNA, luciferase, and sucrose-luciferase right and left panels, respectively.

Translation termination is thought to differ between normal and nonsense-mutated mRNAs. However, no assays to assess translation termination mechanisms *in vivo* are available to date. Because BlaS delays peptide release *in vitro*, we hypothesized that a similar activity of BlaS *in vivo* may resemble delay of termination at a nonsense codon, thus further enhancing assembly of the NMD machinery and possibly decay of an NMD substrate reporter mRNA, or even triggering decay of the corresponding WT mRNA. In contrast, since NMD is a translation-dependent process, inhibition of translation initiation or elongation prevents NMD and stabilizes premature stop codon-containing mRNAs (15,16,52,53). We used our chemiluminescence-based NMD reporter system (29) to investigate (i) whether translation inhibition by BlaS could be observed *in vivo* and (ii) if BlaS treatment exerted a specific effect on the expression of the WT and nonsense-mutated reporter mRNAs. HeLa cells were transfected with reporter constructs comprising a *Renilla* luciferase open reading frame (ORF) N-terminally fused to the human β-globin (HBB) gene with or without a premature stop codon at position 39 in exon 2 of the HBB ORF (*Renilla*-HBB WT/NS39). Co-transfection and co-expression of *Firefly* luciferase served as quantification control of both mRNA and protein expression (29) (Supplementary Figure S5). Messenger RNA levels were quantified by qRT-PCR (Figure 5A). Protein expression was monitored by Western Blot (Supplementary Figure S5A) and by chemiluminescence, measuring enzymatic activity of the produced luciferases (Figure 5B). In HeLa cells transfected with the *Renilla*-HBB NS39 plasmid and treated with BlaS for 3 hours, we observed a BlaS concentration-dependent increase of the reporter mRNA levels (Figure 5A right), whereas levels of the WT mRNA remained unchanged (Figure 5A left). This suggests that inhibition of translation termination by BlaS was either not efficient *in vivo* or failed to simulate the molecular context of termination at a premature stop codon and thus did not lead to mRNA decay.

Interestingly, *Renilla* luciferase enzymatic activity expressed from the nonsense reporter mRNA increased ∼1.8-fold in the presence of 5 and 10 μg/ml BlaS (Figure 5B right) whereas the levels of luciferase expressed from the *Renilla*-HBB WT mRNA had no substantial change (∼1.2-fold, Figure 5B left). At the same time, as judged by the expression of *Firefly* luciferase and actin B protein controls, global translation was not substantially affected at 5–10 μg/ml BlaS (Supplementary Figure S5A). The increased production of the *Renilla*-HBB nonsense protein is consistent with a similarly elevated nonsense mRNA level at 5–10 μg/ml BlaS (Figure 5A right). Thus, the observed increase of both protein and nonsense mRNA levels, indicates that partial inhibition of NMD leads to higher nonsense mRNA level and increased translation of the encoded truncated protein. In contrast, higher concentrations of BlaS (50 and 100 μg/ml BlaS) considerably reduce protein production and enzymatic activity of both the *Renilla*-HBB WT and nonsense reporter while inhibiting NMD as reflected by the up to 4-fold increased expression of the nonsense reporter mRNA up to ∼80% of the WT mRNA level (Figure 5A,B). Taken together, this suggests that stabilization of nonsense mRNAs by BlaS treatment precedes complete translation inhibition.

### BlaS does not prevent nascent chain release *in vivo*

We hypothesized that inhibition of translation termination by BlaS would lead to an accumulation of ribosome-associated nascent protein *in vivo*. In eukaryotes, depending on co-translational folding of the nascent peptide the ribosomal exit tunnel covers ∼30–70 amino acid residues of the growing peptide chain (54). *Firefly* luciferase C-terminally extended by a respective number of amino acid residues can be enzymatically active while still associated with the ribosome whereas luciferase without C-terminal extension is only active after release from the ribosome (55). In our reporter system, the *Renilla* luciferase portion of the *Renilla*-HBB-WT and NS39 proteins contain C-terminal extensions of 149 and 41 amino acids, respectively (29). For β-globin the average peptide length covered by the exit tunnel has been determined in an RRL system to be 30–35 amino acids (56). Therefore, we reasoned that ribosome-bound *Renilla*-HBB-NS39 nascent protein can only be active if translation inhibition occurs at or very close to the premature stop codon. By contrast, *Renilla* luciferase expressed from the *Renilla*-HBB-WT mRNA can metabolize its substrate as soon as the *Renilla* part of the fusion protein has fully emerged from the ribosome and therefore also when translation stalls during elongation within most of the HBB ORF. Using sucrose cushion centrifugation, we investigated the activity of ribosome-associated and released *Renilla* luciferase expressed from *Renilla*-HBB-WT or -NS39 mRNA in the presence of sub-inhibitory or inhibitory concentrations of BlaS (Figure 5C). HeLa cells were transfected with *Renilla*-HBB-WT or *Renilla*-HBB-NS39 encoding plasmids, and the *Firefly* luciferase encoding control plasmid and treated with two concentrations of BlaS (5 and 100 μg/ml) or were left untreated. Cytoplasmic lysates were loaded onto 1 M sucrose cushions, ultracentrifuged and ribosome-containing pellets (P) and ribosome-free supernatant fractions (SNT) were collected to measure the relative luciferase activity (Figure 5C). *Firefly* luciferase that carries no C-terminal extension as expected displayed only background activity in the ribosomal pellet (Supplementary Figure S5C). *Renilla*-HBB WT and -NS39 luciferase activity was significantly reduced when treated with high, inhibitory concentrations of BlaS (Figure 5C) in both ribosome-containing and ribosome-free fractions. By contrast, in the soluble fraction, sub-inhibitory concentrations of BlaS (5 μg/ml) as before resulted in ∼1.8-fold activity increase of *Renilla*-HBB-NS39 relative to the untreated sample (Figure 5C right), while in *Renilla*-HBB WT protein expression remained largely unchanged (Figure 5C left). Notably, the vast majority of both WT and NS39-mutated enzymatically active *Renilla*-HBB protein was found in the ribosome-free fraction, whereas only a minor portion of both were ribosome-associated irrespective of the treatment. This indicates that *in vivo*, BlaS does not efficiently inhibit translation termination and nascent chain release.

## DISCUSSION

Although BlaS was discovered to inhibit protein synthesis as early as in the 1960s, its functional characterization has hitherto been largely limited to bacterial and fungal systems (3–6,10,42,43). We expected BlaS’ mode of action and hence the preferential inhibition of translation termination to be conserved in Mammalia. Importantly, a specific inhibitor of termination would be an attractive tool to study the link between translation termination and NMD *in vivo* in higher eukaryotes. We therefore investigated the mechanism of BlaS-mediated inhibition of mammalian translation biochemically and structurally.

Similar to the situation in bacteria, we found that BlaS slows down peptide release mediated by eRF1, eRF3a and GTP and partially prevents peptidyl-tRNA hydrolysis. At higher concentrations, BlaS inhibits peptide synthesis (Figure 1B), revealing an impact on both elongation and termination steps. However, in our mammalian system, translation termination is impaired at a 6-fold higher concentration than elongation (IC_50_ 21 nM for translation inhibition versus 120 nM for termination inhibition, Figure 1A, C), while the situation in bacteria is the reverse (IC_50_ 182–380 nM versus 32 nM) (3,43). Hence, our *in vitro* experiments indicate that BlaS inhibits elongation more efficiently than peptide release in Mammalia (Figure 1A, C). Consistently, a recent study using an adapted luciferase system to monitor *in vitro* mammalian translation termination in real-time reported that BlaS (at high concentration) strongly inhibits translation elongation (55). However, the authors found no effect on termination (55). We further explored specific steps of termination, and found that stop codon recognition by the eRFs, a precondition of eRF1-mediated peptide release, is not affected by BlaS (Figure 1E, left, Supplementary Figure S1C). UPF3B-mediated ribosome dissociation, which requires peptide release, is impaired in the presence of BlaS (Figure 1E, right), thus confirming that BlaS inhibits the peptide release step during translation termination.

To better understand BlaS’ mode of action regarding mammalian peptide release, we solved the cryo-EM structure of ribosomal termination complexes in the presence of BlaS, eRFs and GTP. Our findings illuminate the differences between the effects of BlaS on bacterial versus mammalian translation. Computational sorting yielded three distinct complexes which all comprised BlaS density bound to the P-loop in the peptidyl transferase center. BlaS displaces both C74 and C75 of the 3′CCA tail from the rRNA (Figure 4A). In contrast, in the bacterial BlaS-ribosome structures only C75 is displaced by ∼7 Å by BlaS which intercalates between C74 and A76 (Figure 4D) (3,10). In our structure, BlaS displaces C74 and C75 up to 14.5 Å resulting in a substantially larger distortion in the peptidyl transferase center (Figure 4A).

Our structures shed light on the mechanism of BlaS’ inhibition of peptide bond formation and peptide release. The displacement of the peptidyl-tRNA from the P-loop towards the A-site is predicted to lead to a series of clashes with the amino acyl-tRNA bound to the A-site (Figure 4B). Accommodation of amino-acyl-tRNA into the A-site would require a conformational adaptation resulting in suboptimal geometry of the tRNAs and thus slowdown of the peptidyl transfer reaction. In the eRF-Bound termination complex, eRF1 is bound in the pre-accommodated state and complexed with eRF3a (Figure 2C). The lower local resolution of the eRFs (4.5-6.0Å) and the flexibility of the P-site tRNA (only weak density for the 3′CCA tail and anticodon stem loop is detected) suggests that these factors are in a state of conformational sampling, likely a product of attempted accommodation of eRF1. Superimposition of the Empty-A structure with a termination complex with bound, accommodated eRF1 (27) reveals a substantial steric clash between the GGQ-loop of eRF1 and the displaced 3′CCA tail of the P-site tRNA (Figure 4C). This indicates that eRF1 accommodation into the peptidyl transferase center is inhibited in the presence of BlaS. In contrast, RF1 accommodation in the bacterial ribosome in the presence of BlaS is enabled due to a ∼2 Å shift of the GGQ-motif of RF1 and a distortion of the conserved U2585 residue (bacterial rRNA numbering) which then binds Gln235 of the GGQ motif (10). Consequently, the interaction between RF1 and peptidyl-tRNA is perturbed and this leads to inhibition of peptide release. In our structures, the 3′CCA tail of P-site tRNA is shifted even further (∼14.5 Å, compared to ∼7 Å) towards the A-site, likely preventing eRF1 accommodation into the 60S A-site (Figure 4C, D).

Because purified ribosomes and translation factors only partially recapitulate the situation in a cellular environment, we used our NMD reporter system (29) to address the impact of a range of BlaS concentrations on translation of a normal and a premature stop codon-mutated transcript in transiently transfected HeLa cells. Specifically, we wondered whether the termination inhibition activity of BlaS would enhance mRNA decay as reflected by both mRNA level and enzymatic reporter activity and thus support a model of NMD where delayed termination triggers mRNA decay (11–13,17). However, while the WT reporter mRNA level remained unaffected by BlaS, the premature stop codon-mutated mRNA level gradually increased with increasing BlaS concentrations to 80% of the WT at the highest concentration tested. At this concentration both WT and truncated protein expression and activity were considerably reduced (Figure 5A, B). NMD inhibition by translation inhibitors is well-described (15,16,52,57–59). It therefore appears that BlaS mainly acts as an elongation inhibitor *in vivo*.

Interestingly, protein expression from *Renilla* luciferase-HBB wildtype and nonsense reporter mRNAs, as measured by enzymatic activity, differed in response to BlaS treatment (Figure 5B): At sub-inhibitory concentrations, wildtype protein expression was virtually unaffected by BlaS and only inhibited at high concentrations. In contrast, sub-inhibitory concentrations of BlaS induced an increase of both expression and activity of the truncated reporter protein (Figure 5B, right panel; Supplementary Figure S5), while global translation seems not to be affected under these conditions. Our sucrose centrifugation experiments revealed that neither full-length nor truncated *Renilla*-HBB nascent chains accumulate on elongation- or termination-stalled ribosomes after BlaS treatment. It thus appears that either termination is not significantly inhibited at these BlaS concentrations or that *in vivo* stalled ribosomes are rapidly dissolved and the produced nonsense proteins are released from the ribosome (Figure 5C). We note that the stimulatory effect on nonsense protein expression at sub-inhibitory BlaS concentrations appears to be proportional to the stabilization of the nonsense mRNA levels (Figure 5A, B); both mRNA and protein enzymatic activity increased ∼1.8-fold at 5 and 10 μg/ml BlaS. In conclusion, NMD appears to be sensitive to small disturbances of translation dynamics by antibiotics.

In recent years, translation inhibitors were used to study no-go decay and ribosome quality control mechanisms (37,60). Subinhibitory cycloheximide concentrations induce ribosome collisions and trigger no-go quality control (60). Ribosome collisions induced by difficult-to-translate sequences (e.g. by poly(A) sequences) and subsequent translational quality control can be prevented by sub-inhibitory amounts of initiation inhibitor pactamycin leading to a lower ribosome density on the mRNA transcript; and this slowdown of translation through a problematic sequence does not cause ribosome collisions (37). Here, the sub-inhibitory BlaS concentrations are likely to slow-down elongation by binding and dissociating from the ribosome. At the same time, this mildly impaired elongation may prevent translation termination at the premature stop codon from being recognized as slow and aberrant, and therefore NMD is not triggered. It seems that by generating a translation problem elsewhere (during elongation), problems during termination can be avoided, possibly by reducing ribosomal collisions at the premature stop codon. This suggests that the NMD machinery can be impaired by the absence of active translation (global translation inhibition) as well as by changed translation dynamics (slowed elongation).

Further investigation is required to understand how cells use such translation dynamics to finetune the levels of endogenous NMD-target mRNAs. Enhanced expression of proteins encoded by exogenous and endogenous NMD target mRNAs has been observed upon treatment with other NMD inhibitors (59). Moreover, the extent of nonsense mRNA stabilization in response to antibiotics treatment varies depending on NMD substrate and cell type (61,62). Further studies will reveal if such antibiotics-induced effects could be exploited for new treatment strategies of NMD-associated diseases, where NMD aggravates the disease phenotype and the C-terminally truncated nonsense protein would be (partially) functional.

## DATA AVAILABILITY

The ribosome maps have been deposited to the EMDB with accession codes EMD-12633 (Empty-A), EMD-12631 (Hybrid) and EMD-12632 (eRF-Bound). Atomic coordinates have been deposited to the Protein Data Bank under accession codes PDB 7NWI (Empty-A), 7NWG (Hybrid) and 7NWH (eRF-Bound). All other data are available upon request.

## Supplementary Material

gkab532_Supplemental_File
