# Supplementary material for: Blasticidin S inhibits mammalian translation and enhances production of protein encoded by nonsense mRNA
Source: Nucleic Acids Res. 2021 Jun 22;49(13):7665–79. doi: 10.1093/nar/gkab532 (PMC8287960; doi:10.1093/nar/gkab532)
Supplement: gkab532_Supplemental_File [file gkab532_supplemental_file.docx]

**Supplementary Material**

**Blasticidin S inhibits mammalian translation and enhances production of protein encoded by nonsense mRNA**

Authors:

Kyle T. Powers^1†^, Flint Stevenson-Jones^1†^, Sathish K.N. Yadav^1†^, Beate Amthor^2^,^3^, Joshua C. Bufton^1^, Ufuk Borucu^1^, Dakang Shen^1^, Jonas P. Becker^2,3^, Daria Lavysh^2,3^, Matthias W. Hentze^3,4^, Andreas E. Kulozik^2,3^, Gabriele Neu-Yilik,^2,3^*, Christiane Schaffitzel^1^*

^†^ These authors contributed equally to this work: Kyle T. Powers, Flint Stevenson-Jones, Sathish K.N. Yadav

Affiliations:

^1^ University of Bristol, School of Biochemistry, University Walk, Bristol BS8 1TD, United Kingdom.

^2^ Department of Pediatric Oncology, Hematology and Immunology, Hopp Children's Cancer Research Center Heidelberg (KiTZ), University of Heidelberg, Heidelberg, Germany.

^3^ Molecular Medicine Partnership Unit (MMPU) European Molecular Biology Laboratory (EMBL) and University of Heidelberg, Heidelberg

^4^ European Molecular Biology Laboratory (EMBL), Heidelberg

* Correspondence should be addressed to CS (christiane.berger-schaffitzel@bristol.ac.uk) and GNY ([gabriele.neu-yilik@med.uni-heidelberg.de](mailto:gabriele.neu-yilik@med.uni-heidelberg.de))


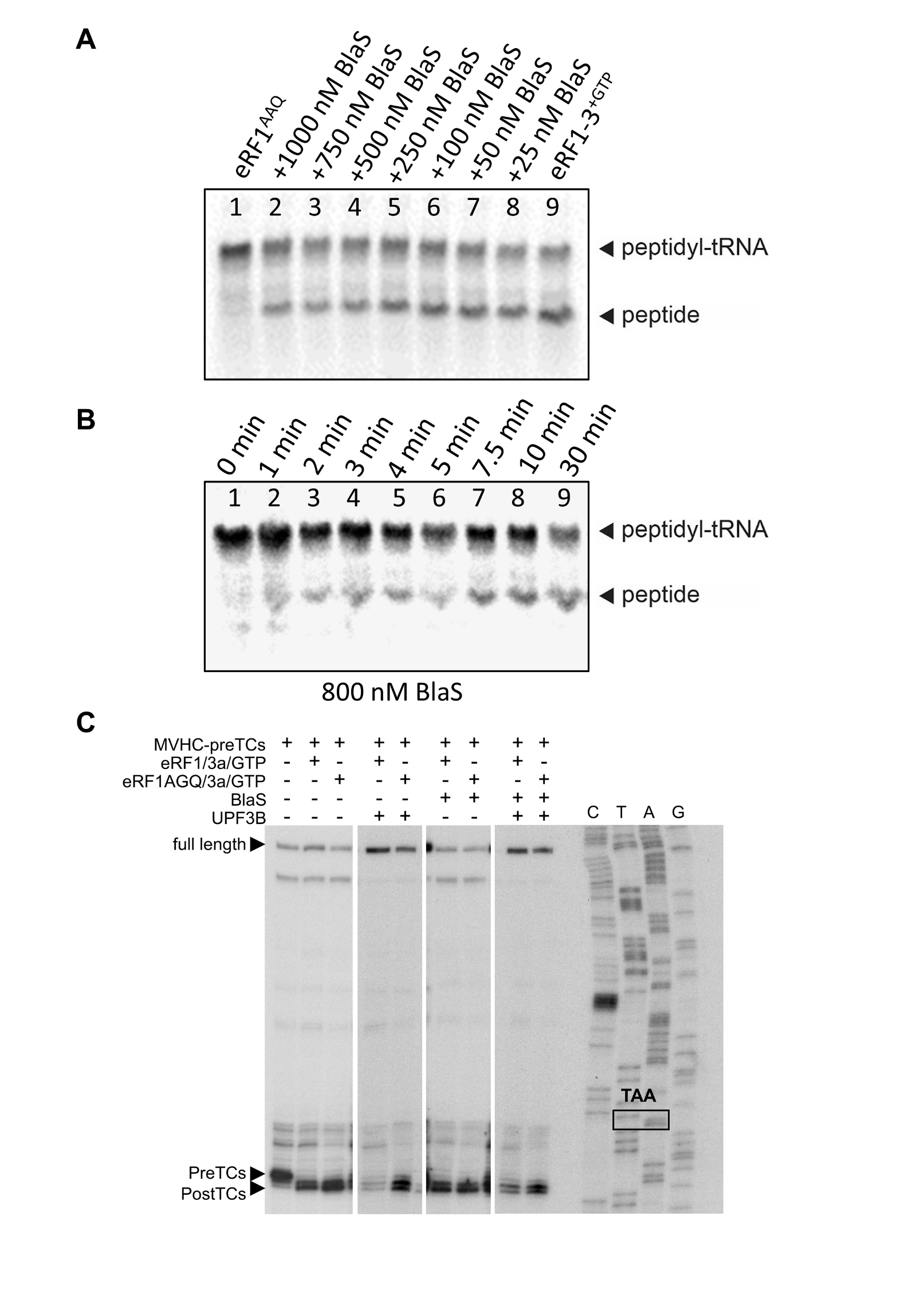


**Supplementary Figure 1: Impact of BlaS on translation elongation and peptide release. A,** Representative autoradiography of [^35^S]-methionine labelled 3xFLAG-Sec61β-VHP. Translation reaction was performed for 30 min in RRL, and [^35^S]-methionine labelled products were analyzed by gel electrophoresis and autoradiography. The proportion of peptide release was determined by assessment of the relative amounts of peptidyl-tRNA versus free peptide (plotted in Fig. 1B). Concentrations of supplemented BlaS are indicated. Pre-TC’s prepared with eRF1^AAQ^ (lane 1) provide a control for no peptide release. Pre-TC’s supplemented with wild-type release factors eRF1 and eRF3a with GTP (positive control: no BlaS, lane 9) indicate a background of ~45% of peptides not released under our experimental conditions. **B,** Time course of peptide release in the presence of 800 nM BlaS. Reaction times are indicated. 0 min (lane 1) served as the reference for no release. **C,** Toeprinting analysis of ribosomal complexes obtained by incubating preTC assembled on MVHC-stop mRNA (MVHC-preTCs) with eRF1/eRF3a and GTP, or eRF1^AGQ^/eRF3a and GTP in the presence of 5 µg/mL BlaS or UPF3B or both.


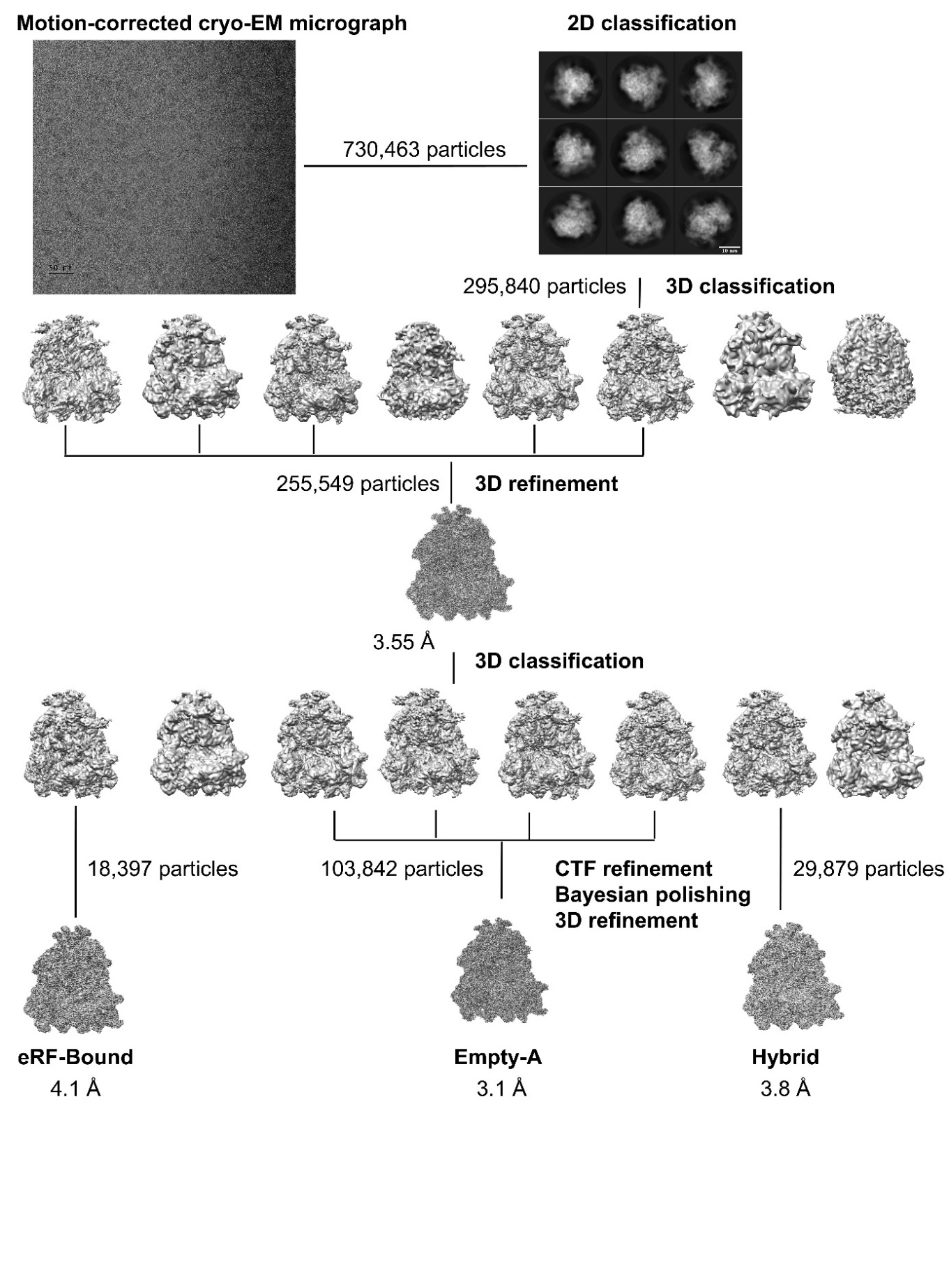


**Supplementary Figure 2**. **Cryo-EM image processing workflow**. A motion-corrected cryo-EM micrograph is shown (scale bar 50 nm), reference-free 2D class averages (scale bar 10 nm), 3D classification and 3D refinement resulting in 3 cryo-EM maps corresponding Empty-A, Hybrid and eRF-Bound.

**
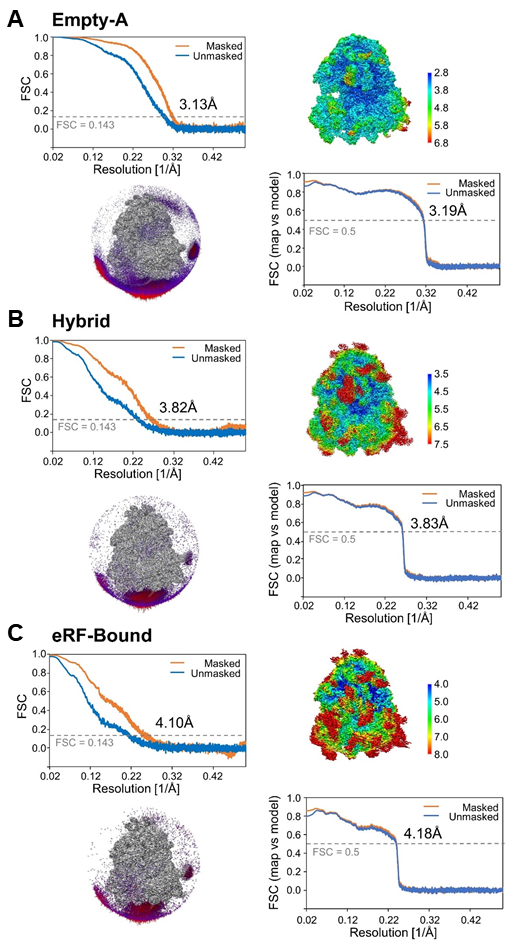
**

**Supplementary Figure 3.** **Cryo-EM structure validation.** Top left: Fourier Shell Correlation (FSC) curve after gold standard refinement. Top right: Cryo-EM reconstructed map colored according to the local resolution. Below left: Orientation distribution of views that contributed to this map. Longer red rods represent orientations that comprise more particles. Below right: Cross-validation FSC curves for the refined model versus the final masked and unmasked maps, shown for (**A**) the Empty-A map (**B**) the Hybrid map and (**C**) the eRF-Bound map.


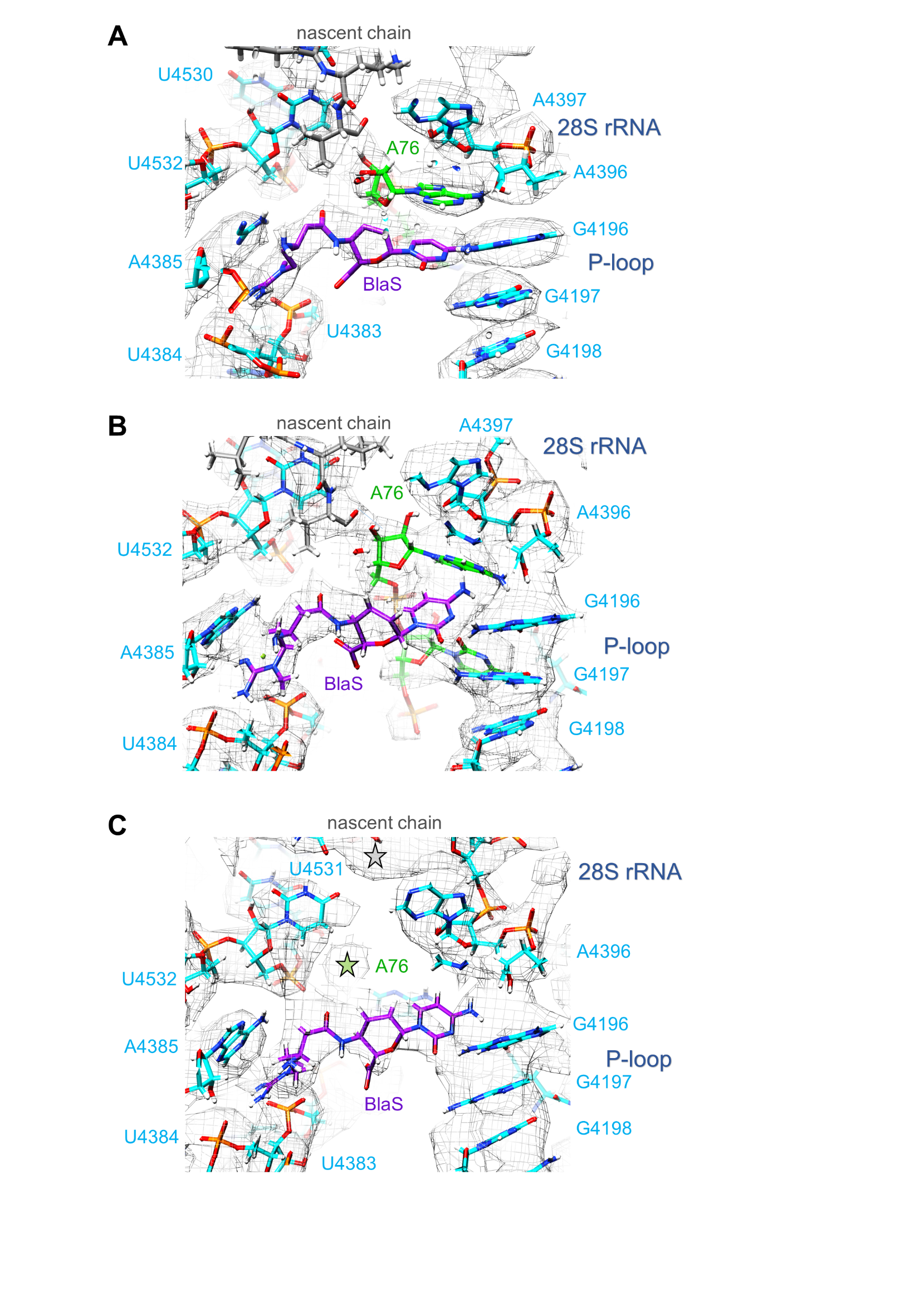


**Supplementary Figure 4. BlaS binding to the P-site in the 60S peptidyl transferase center.** The EM density of BlaS and the residues in the peptidyl transferase center are shown in a clipped view for the Empty-A structure (**A**), the Hybrid structure (**B**) and eRF-Bound structure (**C**). EM density is depicted as a mesh colored in grey. BlaS is depicted in sticks colored in purple, 28S rRNA in cyan, tRNA in green and nascent chain in grey. The stars in panel C highlight the EM density for peptidyl-tRNA which was not modelled.

**
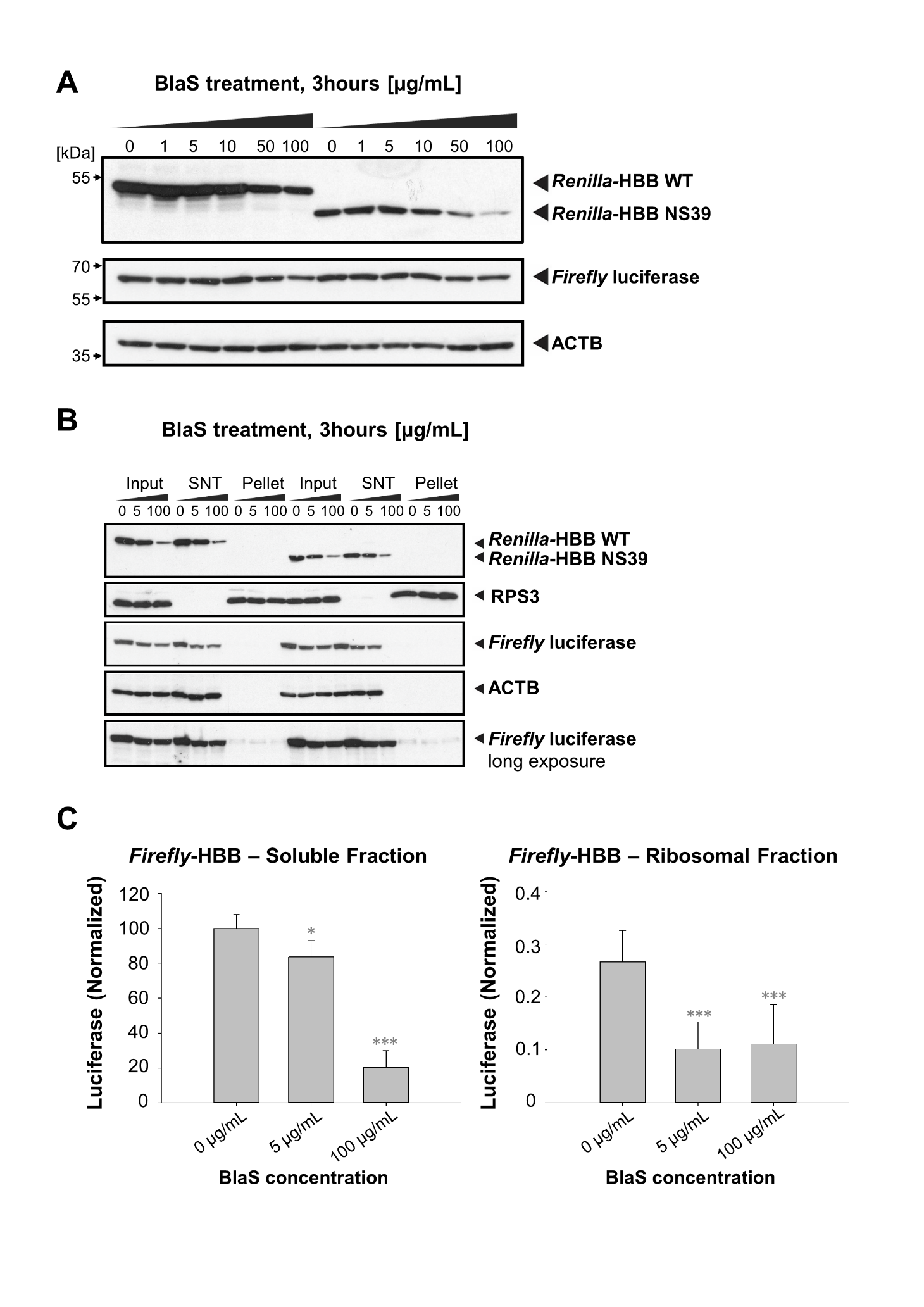
**

**Supplementary Figure 5: Expression of *Renilla-*HBB-WT and C-terminally truncated *Renilla-*HBB-NS39 protein in the presence of BlaS. A,** Above: Detection of *Renilla*-HBB WT (left, lanes 1-6) and *Renilla*-HBB NS39 (right, lanes 7-12) expressed in the presence of indicated concentrations of BlaS and using an anti-*Renilla* Luciferase antibody. Middle: Control detecting expression of co-transfected *Firefly* luciferase using an anti-*Firefly* Luciferase antibody in the presence of indicated concentrations of BlaS. Below: Loading control detecting endogenous actin beta (ACTB). **B,** Western Blots detecting *Renilla*-HBB WT, *Renilla*-HBB NS39, *Firefly* luciferase, actin beta and ribosomal protein S3 in the input (before centrifugation), the supernatant fraction (SNT) and the ribosomal pellet after sucrose cushion centrifugation. The concentration of BlaS added during expression is indicated. **C,** Control *Firefly* luciferase activity measurements following treatment of transfected cells with indicated concentrations of BlaS and sucrose cushion centrifugation. Left: Luciferase enzymatic activities in the supernatant fraction. Right: Luciferase activities in the ribosomal pellet fraction. Each bar indicates average with SD from three or more measurements. One-way ANOVAs using the Holm-Šidák statistical significance tests (α=0.05) are indicated with asterisks identifying those with a p value 0.01 < p $\leq$ 0.05 having one, 0.001 < p $\leq$ 0.01 two, and three asterisks for p $\leq$ 0.001. All luciferase activities are normalized towards the activity of luciferase in the supernatant fraction of the untreated sample (0 µg/mL BlaS).

**Supplementary Table 1. Cryo-EM data collection and refinement statistics.**

|  | **Empty-A** | **eRF-Bound** | **Hybrid** |
| --- | --- | --- | --- |
| Voltage (kV) | 200 | 200 | 200 |
| Magnification (nominal) | 100,000 | 100,000 | 100,000 |
| Pixel size (Å/pix) | 1.35 (0.675) | 1.35 (0.675) | 1.35 (0.675) |
| Flux (e^-^/pix/sec) | 9.6 | 9.6 | 9.6 |
| Frames per exposure | 40 | 40 | 40 |
| Exposure (e^-^/Å^2^) | 41.9 | 41.9 | 41.9 |
| Defocus range (μm) | -0.4 to -2 | -0.4 to -2 | -0.4 to -2 |
| Micrographs collected | 6200 | 6200 | 6200 |
| Particles final | 103,842 | 18,397 | 29,879 |
| Map sharpening B-factor (Å^2^) | -50 | -25 | -25 |
| Masked resolution (Å) 0.143 FSC | 3.13 | 4.1 | 3.82 |

**Refinement**

|  | **Empty-A** | **eRF-Bound** | **Hybrid** |
| --- | --- | --- | --- |
| Composition | | | |
| Non-hydrogen atoms | 216,929 | 219,378 | 220,150 |
| Nucleotides | 11,505 | 12,540 | 11,877 |
| RNA bases | 5,743 | 5,527 | 5,813 |
| Ligands (BlaS/Mg^2+^/Zn^2+^) | 1/200/5 | 1/281/8 | 1/300/8 |
| RMSD bonds (Å) | 0.01 | 0.014 | 0.013 |
| RMSD angles (º) | 1.06 | 1.531 | 1.437 |
| Mean B-factors (Å^2^) | | | |
| Protein Residues | 48.68 | 39.19 | 88.42 |
| Nucleotides | 66.34 | 45.33 | 101.17 |
| Ligands (BlaS) | 41.16 | 26.65 | 57.31 |
| Ramachandran | | | |
| Favored (%) | 88.09 | 92.72 | 88.96 |
| Allowed (%) | 11.76 | 6.84 | 10.65 |
| Outliers (%) | 0.15 | 0.44 | 0.38 |
| Rotamer favored (%) | 88.09 | 92.07 | 94.62 |
| Rotamer outliers (%) | 0.01 | 0.73 | 0.40 |
| Validation (RNA) | | | |
| Correct sugar puckers (%) | 98.38 | 96.65 | 98.45 |
| Good backbones (%) | 70.68 | 75.66 | 66.02 |
| MolProbity score | 1.72 | 2.31 | 2.39 |
| Model resolution (Å) at 0.500 FSC | 3.19 | 4.18 | 3.83 |
